# Supplementary material for: Impact on alcohol selection and online purchasing of changing the proportion of available non-alcoholic versus alcoholic drinks: A randomised controlled trial
Source: PLoS Med. 2023 Mar 30;20(3):e1004193. doi: 10.1371/journal.pmed.1004193 (PMC10062674; doi:10.1371/journal.pmed.1004193)
Supplement: S1 Supporting information — Table A. Proportions of non-alcoholic and alcoholic drinks displayed in the selection task. Table B. Drink options used in the selection task. Table C. Number of zero values in the selection task by group and outcome. Table D. Effect of attrition from selection to purchasing on groups. Table E. Effect of attrition from selection to purchasing on weekly units purchased at baseline. Table F. Additional outcomes: Raw means. Table G. Full model results for additional outcomes. (DOCX) [file pmed.1004193.s001.docx]

**Impact on alcohol selection and online purchasing of changing the proportion of available non-alcoholic versus alcoholic drinks: A randomised controlled trial**

*Supplementary Material*

**Drink options and detailed task description**

Alcohol-free beer, cider and wine were clearly labelled to ensure they were not confused with alcoholic drinks. Each drink option presented was a different brand, i.e., as the availability of non-alcoholic drink options increased, there was a larger choice of brands from which to choose. Drink images were all shown as bottles or cans, either as single items or multipacks, depending on the availability of products at Tesco online supermarket. Price promotions and variations for all drinks included in the selection task were checked every month via Tesco.com and recorded. Prices shown in the task therefore reflected the full price on Tesco.com for the duration of the study.

**Table A. Proportions of non-alcoholic and alcoholic drinks displayed in the selection task**

|  | **Drink subset availability** | | |
| --- | --- | --- | --- |
| **Drink range** | **Group 1: 25% non-alcoholic** | **Group 2: 50% non-alcoholic** | **Group 3: 75% non-alcoholic** |
| Beer, cider and soft drinks (n) | *AF beer and cider (4)*  AF lager (2)  AF ale (1)  AF cider (1)  *Soft drinks (4)*    *Beer and cider (24)*  Lager (10)  Ale (8)  Cider (6) | *AF beer and cider (8)*  AF lager (3)  AF ale (3)  AF cider (2)  *Soft drinks (8)*  *Beer and cider (16)*  Lager (6)  Ale (6)  Cider (4) | *Alcohol-free (AF) beer and cider (12)*  AF lager (4)  AF ale (4)  AF cider (4)  *Soft drinks (12)*  *Beer and cider (8)*  Lager (3)  Ale (3)  Cider (2) |
| Wine and soft drinks (n) | *AF wine (4)*  AF red wine (1)  AF white wine (1)  AF rose or sparkling wine (2)  *Soft drinks (4)*    W*ine (24)*  Red wine (9)  White wine (9)  Rosé or sparkling wine (6) | *AF wine (8)*  AF red wine (3)  AF white wine (3)  AF rose or sparkling wine (2)  *Soft drinks (8)*    W*ine (16)*  Red wine (6)  White wine (6)  Rosé or sparkling wine (4) | *AF wine (12)*  AF red wine (3)  AF white wine (5)  AF rose or sparkling wine (4)  *Soft drinks (12)*    *Wine (8)*  Red wine (3)  White wine (3)  Rosé or sparkling wine (2) |

*AF = Alcohol Free*

**Table B. Drink options used in the selection task (prices based on Tesco.com, January 2021). Products shown in bold are those used in the 50% non-alcoholic condition.**

| **Brand name** | **ABV** | **Volume** | **Price** | **Alcohol / alcohol-free match** |
| --- | --- | --- | --- | --- |
| ***Alcohol-free beer*** | | | | |
| **Heineken Alcohol Free Beer** | **0.00%** | **12x330ml** | **£8.00 (£2.03/l)** | **Y** |
| **Peroni Liberia Alcohol Free Bottle** | **0.00%** | **4x330ml** | **£4.50 (£3.41/l)** | **Y** |
| **San Miguel 0.0% Alcohol Free Lager** | **0.00%** | **4x330ml** | **£3.50 (£2.66/l)** | **Y** |
| Becks Blue Alcohol Free | 0.05% | 15x275ml | £7.00 (£1.70/l) | Brand only (+ exposure) |
| **Brewdog Punk Af** | **0.50%** | **4x330ml** | **£4.50 (£3.41/l)** | **Y** |
| **Hoegaarden Wit Blanche Wheat Beer 0.0** | **0.00%** | **4x330ml** | **£4.00 (£3.04/l)** | **Y** |
| **Adnams Ghost Ship Bottle Beer 0.5%** | **0.50%** | **500ml** | **£1.30 (£2.60/l)** | **Y** |
| Doom Bar Zero Amber Ale | 0.00% | 500ml | £1.30 (£2.60/l) | Y |
| ***Alcohol-free Cider*** | | | | |
| **Friels Low Alcohol Cider** | **0.50%** | **4x330ml** | **£3.50 (£2.66/l);** | **Y** |
| **Kopparberg Premium Cider Mixed Fruit Alcohol Free** | **0.05%** | **4x330ml** | **£3.50 (£2.66/l)** | **Y** |
| Stowford Press Apple Cider Low Alcohol | 0.50% | 500ml | £1.30 (£2.60/l) | Brand only |
| Kopparberg Alcohol Free Pear Cider | 0.05% | 500ml | £1.30 (£2.60/l) | Y |
| ***Alcohol-free wine*** | | | | |
| **Lindeman's Alcohol Free Cabernet Sauvignon** | **0.50%** | **750ml** | **£4.00** | **Brand only** |
| **Tesco Low Alcohol Cabernet Tempranillo** | **0.50%** | **750ml** | **£2.75** | **Y** |
| **Eisberg Merlot Alcohol Free Wine** | **0.00%** | **750ml** | **£3.50** | **Grape only** |
| **Hardys Alcohol Free Chardonnay** | **0.05%** | **750ml** | **£5.00** | **Y** |
| **Lindeman's Alcohol Free Semillon Chardonnay** | **0.50%** | **750ml** | **£4.00** | **Brand only** |
| **Tesco Low Alcohol Sauvignon Blanc** | **0.50%** | **750ml** | **£2.75** | **Y** |
| Eisberg Sauvignon Alcohol Free | 0.00% | 750ml | £3.50 | Grape only |
| Mcguigan Zero Alcohol Free Sauvignon Blanc | 0.05% | 750ml | £5.00 | Brand only |
| **Tesco Low Alcohol Garnacha Rose** | **0.50%** | **750ml** | **£2.75** | **Y** |
| Eisberg Rose Alcohol Free | 0.00% | 750ml | £3.50 | Type only |
| **Freixenet 0.0% Alcohol Free Sparkling** | **0.00%** | **750ml** | **£5.00** | **Y** |
| Tesco Low Alcohol Sparkling White Wine | 0.50% | 750ml | £2.75 | Y |
| ***Soft drinks***^[[1]](#footnote-1)^ ***(used in alcohol-free beer, cider and soft drinks selection)*** | | | | |
| **Fentimans Curiosity Cola** | **n/a** | **4x275ml** | **£4.53 (£0.41/100ml)** | **n/a** |
| **San Pellegrino Sparkling Water** | **n/a** | **6 x 1l** | **£5.50 (£0.09/100ml)** | **n/a** |
| **Belvoir Light Elderflower Presse** | **n/a** | **750ml** | **£2.49 (£0.33/100ml)** | **n/a** |
| **San Pellegrino Sparkling Limonata** | **n/a** | **6X330ml** | **£3.79 (£0.19/100ml)** | **n/a** |
| **J20 Orange & Passion Fruit** | **n/a** | **6X275Ml** | **£6.00 (£0.36/100ml)** | **n/a** |
| **Tesco Soda Water** | **n/a** | **1l** | **£0.50 (£0.05/100ml)** | **n/a** |
| **Schweppes Tonic Water** | **n/a** | **12 X 150ml** | **£4.00 (£0.22/100ml)** | **n/a** |
| **Fentimans Traditional Ginger Beer** | **n/a** | **4x275ml** | **£4.53 (£0.41/100ml)** | **n/a** |
| Appletiser 100% Sparkling Apple Juice | n/a | 750ml | £1.45 (£0.19/100ml) | n/a |
| Lipton Ice Tea Peach Flavour | n/a | 1.25l | £1.60 (£0.13/100ml) | n/a |
| Tesco Low Calorie Indian Tonic Water Cans | n/a | 6X250ml | £2.25 (£0.15/100ml) | n/a |
| Tesco Low Calorie Ginger Ale | n/a | 1l | £0.50 (£0.05/100ml) | n/a |
| ***Soft drinks (used in alcohol-free wine and soft drinks selection)*** | | | | |
| **J2O Spritz Apple Watermelon** | **n/a** | **6X275ml** | **£6.00 (£0.36/100ml)** | **n/a** |
| **Shloer Sparkling White Grape Juice** | **n/a** | **750ml** | **£2.20 (£0.29/100ml)** | **n/a** |
| **Fentimans Traditional Rose Lemonade** | **n/a** | **750ml** | **£2.95 (£0.39/100ml)** | **n/a** |
| **San Pellegrino Aranciata Rossa 6 Pack Can** | **n/a** | **6X330ml** | **£3.79 (£0.19/100ml)** | **n/a** |
| **Oasis Summer Fruit** | **n/a** | **1.5l** | **£1.00 (£0.07/100ml)** | **n/a** |
| **Schweppes Soda Water** | **n/a** | **1l** | **£1.50 (£0.15/100ml)** | **n/a** |
| **London Essence Orange & Elderflower Tonic** | **n/a** | **6X150ml** | **£3.25 (£0.36/100ml)** | **n/a** |
| **Schweppes Canada Dry Ginger Ale** | **n/a** | **1l** | **£1.50 (£0.15/100ml)** | **n/a** |
| Bottlegreen Elderflower Presse | n/a | 750ml | £2.49 (£0.33/100ml) | n/a |
| J20 Apple & Raspberry | n/a | 6x275ml | £6.00 (£0.36/100ml) | n/a |
| Fever-Tree Indian Tonic Water | n/a | 8 X 150Ml | £4.25 (£0.36/100ml) | n/a |
| Tesco No Added Sugar Ginger Beer | n/a | 4 X 330Ml | £0.99 (£0.08/100ml) | n/a |
| ***Beer*** | | | | |
| **Heineken** | **5.00%** | **12x330ml** | **£11.00 (£2.78/l)** | **Y** |
| **Peroni Nastro Azzurro** | **5.10%** | **4x330ml** | **£6.50 (£4.93/l)** | **Y** |
| **San Miguel** | **5.00%** | **4x330ml** | **£4.50 (£3.41/l)** | **Y** |
| **Becks Lager Beer** | **4.00%** | **20x275ml** | **£10.00 (£1.82/l)** | **Larger multipack** |
| **Budweiser** | **4.50%** | **15x440ml** | **£12.00 (£1.82/l)** | **Addition** |
| **Stella Artois Premium Lager** | **4.60%** | **4x568ml** | **£5.39 (£2.38/l)** | **Addition** |
| Fosters | 4.00% | 4x440ml | £3.49 (£1.99/l) | Addition |
| Carling Lager | 4.00% | 18x440ml | £9.99 (£1.27/l) | Addition |
| Amstel Lager Beer Can | 4.10% | 4x440ml | £4.00 (£2.28/l) | Addition |
| Guinness Draught | 4.10% | 4x440ml | £4.49 (£2.56/l) | Addition |
| **BrewDog Punk Ipa** | **5.40%** | **4x330ml** | **£6.00 (£4.55/l)** | **Y** |
| **Hoegaarden White Beer** | **4.90%** | **4x330ml** | **£4.50 (£3.41/l)** | **Y** |
| **Adnams Ghost Ship** | **4.30%** | **500ml** | **£1.70 (£3.40/l)** | **Y** |
| **Sharps Doom Bar** | **4.30%** | **500ml** | **£1.49 (£2.98/l)** | **Y** |
| **Hobgoblin Ipa** | **5.30%** | **500ml** | **£1.70 (£3.40/l)** | **Addition** |
| **Old Speckled Hen Can** | **5.00%** | **4x500ml** | **£4.29 (£2.15/l)** | **Addition** |
| Abbot Ale Strong Bitter | 5.00% | 4x500ml | £4.49 (£2.25/l) | Addition |
| Fullers London Pride | 4.70% | 500ml | £1.70 (£3.40/l) | Addition |
| ***Cider*** | | | | |
| **Friels Vintage Cider** | **7.40%** | **4x330ml** | **£4.50 (£3.41/l)** | **Y** |
| **Kopparberg Mixed Fruit Cider** | **4.00%** | **4x330ml** | **£5.00 (£3.79/l)** | **Y** |
| **Stowford Press Apple Cider** | **4.50%** | **4x440ml** | **£3.50 (£1.99/l)** | **Multipack vs single** |
| **Kopparberg Pear** | **4.50%** | **500ml** | **£2.00 (£4.00/l)** | **Y** |
| Strongbow Original Cider | 4.50% | 4x440ml | £4.00 (£2.28/l) | Addition |
| Bulmers Original Premium Cider | 4.50% | 8x500ml | £6.00 (£1.50/l) | Addition |
| ***Wine*** | | | | |
| **Lindeman's Bin 50 Shiraz** | **13.50%** | **750ml** | **£7.00** | **Brand only** |
| **Tesco Spanish Tempranillo** | **12.00%** | **750ml** | **£3.69** | **Similar** |
| **Hardys Varietal Range Merlot** | **13.00%** | **750ml** | **£6.00** | **Grape only** |
| **Yellow Tail Pinot Noir** | **13.50%** | **750ml** | **£7.00** | **Addition** |
| **Gallo Family Vineyards Merlot** | **13.00%** | **750ml** | **£6.00** | **Addition** |
| **Wolf Blass Yellow Label Cabernet Sauvignon** | **13.50%** | **750ml** | **£8.00** | **Addition** |
| Campo Viejo Rioja Garnacha | 14.00% | 750ml | £8.00 | Addition |
| Kumala Reserve Malbec | 13.50% | 750ml | £7.00 | Addition |
| Mcguigan Reserve Cabernet | 12.00% | 750ml | £7.00 | Addition |
| **Hardys Crest Chardonnay** | **13.00%** | **750ml** | **£7.00** | **Y** |
| **Lindeman's Bin 65 Chardonnay** | **12.50%** | **750ml** | **£7.00** | **Similar** |
| **Wolf Blass Yellow Label Sauvignon Blanc** | **13.00%** | **750ml** | **£8.00** | **Grape only** |
| **Mcguigan Reserve Chardonnay** | **12.50%** | **750ml** | **£7.00** | **Brand only** |
| **Tesco Finest Marlborough Sauvignon Blanc** | **12.50%** | **750ml** | **£8.00** | **Y** |
| **Yellow Tail Pinot Grigio** | **11.50%** | **750ml** | **£7.00** | **Addition** |
| First Cape Special Cuvee Chenin Blanc | 12.50% | 750ml | £6.00 | Addition |
| Isla Negra Sauvignon Blanc Px | 12.00% | 750ml | £5.00 | Addition |
| Kumala Reserve Chenin Blanc | 13.00% | 750ml | £7.00 | Addition |
| **Tesco Tempranillo Garnacha Rose** | **11.50%** | **750ml** | **£4.50** | **Y** |
| **Blossom Hill White Zinfandel** | **11.00%** | **750ml** | **£6.00** | **Type only** |
| Yellow Tail Rose | 12.00% | 750ml | £7.00 | Addition |
| **Freixenet Prosecco Doc** | **11.00%** | **750ml** | **£12.00** | **Y** |
| **Tesco Finest Prosecco Doc** | **11.00%** | **750ml** | **£8.00** | **Y** |
| Finest Prosecco Valdobbiadene Docg | 11.50% | 750ml | £10.00 | Addition |

**Table C. Number of zero values in the selection task by group and outcome.**

| **Number of participants that:** | **Group 1: 25% non-alcoholic**  **(n = 207)** | **Group 2: 50% non-alcoholic**  **(n = 194)** | **Group 3: 75% non-alcoholic**  **(n = 206)** |
| --- | --- | --- | --- |
| Selected no drinks (alcoholic or non-alcoholic) | 2 | 4 | 5 |
| Selected no alcoholic drinks | 12 | 18 | 37 |
| Selected no non-alcoholic drinks | 120 | 72 | 56 |
| Selected no drinks containing alcohol units | 7 | 14 | 27 |

**Secondary outcomes**

A substantial number of datapoints met our pre-specified criterion of being possible outliers (Median Absolute Deviation (MAD) of >3) but these were typically considered to the part of the natural variability of such selection and purchasing measurements [1]. Due to concerns about unreasonable influence of any extreme or highly improbable values, we excluded one extreme value for units of alcohol purchased with a MAD of >15 (being more than two times the next highest value), with sales of over 290 units of alcohol (more than 50% more than the next highest value seen - 192.45 vs 291.85 - and relative to typical baseline purchasing and consumption by the same individual of only 0-2 units, and that worsened model fit.

**Effect of attrition**

**Table D: Effect of attrition from selection to purchasing on groups (n, [%])**

|  | **Group 1: 25% non-alcoholic** | **Group 2: 50% non-alcoholic** | **Group 3: 75% non-alcoholic** | **Chi-squared**  **p-value** |
| --- | --- | --- | --- | --- |
| Primary outcome: Selected  n=607 | 207 [100] | 194 [100] | 206 [100] | - |
| Secondary outcome:  Purchased  n=422 | 145 [70] | 141 [73] | 136 [66] | 0.344 |
| Per-protocol 1  Primary outcome: Selected  n=344 | 120 [58] | 115 [59] | 109 [52] | 0.394 |
| Per-protocol 2  Primary outcome: Selected  n=182 | 67 [32] | 62 [32] | 53 [26] | 0.260 |

**Table E: Effect of attrition from selection to purchasing on weekly units purchased at baseline (mean [median])**

|  | **Group 1: 25% non-alcoholic** | **Group 2: 50% non-alcoholic** | **Group 3: 75% non-alcoholic** | **Model**  **p-value*:**  **Attrition (interaction)** |
| --- | --- | --- | --- | --- |
| Primary outcome: Selected  n=607 | 40.6 [32.6] | 36.8 [29.3] | 40.9 [34.6] |  |
| Secondary outcome:  Purchased  n=422 | 36.39 [32.6] | 37.5 [27.5] | 38.2 [31.9] | 0.125 (0.300) |
| Per-protocol 1  Primary outcome: Selected  n=344 | 34.7 [31.58] | 36.7 [27.5] | 36.7 [30.0] | 0.017 (0.396) |
| Per-protocol 2  Primary outcome: Selected  n=182 | 34.1 [28.0] | 34.7 [27.8] | 31.3 [27.2] | 0.024 (0.440) |

*A model using sqrt (weekly units purchased) had acceptable regression diagnostics.

**Additional outcomes**

When including additional drinks from study categories only, those in the 75% non-alcoholic group purchased fewer alcoholic drinks than those in the 50% non-alcoholic group (-25%; 95%CIs -41%, -6%; p = .015). There was no evidence of a difference between the 75% non-alcoholic and 25% non-alcoholic groups (-18%; 95%CIs -35%,4%; p = .103), or the 25% non-alcoholic and 50% non-alcoholic groups (10%; 95%CIs -12%,39%; p = .402). There was evidence that those in the 75% non-alcoholic group purchased more non-alcoholic drinks than those in the 50% non-alcoholic group (66%; 95%CIs 10%,149%; p = .014) and the 25% non-alcoholic group (78%; 95%CIs 18%,166%; p = .005). There was no evidence of a difference between the 25% non-alcoholic and 50% non-alcoholic groups (7%; 95%CIs -28%,60%; p = .734).

When including all additional drinks, there was no evidence of a difference between groups for alcoholic drinks purchased, although there were non-significant reductions between the 75% non-alcoholic and 25% non-alcoholic groups (-16%; 95%CIs -32%, 6%; p = .141), and the 75% non-alcoholic and Equal groups (-24%; 95%CIs -39%, -4%; p = .022). There was evidence of an increase in non-alcoholic drinks purchased between the 75% non-alcoholic and 25% non-alcoholic groups (60%; 95%CI 11%, 132%; p = .011), and non-significant increases between the 50% non-alcoholic and the 75% non-alcoholic groups (28%) and the 50% non-alcoholic and the 25% non-alcoholic groups (25%).

There was no evidence that the total number of drinks selected or purchased differed between groups.

**Table F. Additional outcomes (raw means (SD))**

|  | **GROUP 1: 25% non-alcoholic**  **(n = 207)** | **GROUP 2: 50% non-alcoholic**  **(n = 194)** | **GROUP 3: 75% non-alcoholic**  **(n = 206)** |
| --- | --- | --- | --- |
|  | **Mean (SD)** | | |
| **Selection** | | | |
| Total number of drinks selected | 16.0 (21.4) | 15.2 (16.2) | 15.2 (18.8) |
| **Secondary and additional outcomes – purchasing** | | | |
|  | **GROUP 1: 25% non-alcoholic**  **(n = 145)** | **GROUP 2: 50% non-alcoholic**  **(n = 141)** | **GROUP 3: 75% non-alcoholic**  **(n = 136)** |
| Total number of drinks purchased (including additional drinks from study categories only) | 13.27 (13.59) | 14.17 (13.65) | 14.71 (12.05) |
| Total number of drinks purchased (including all additional drinks) | 15.42 (14.62) | 17.45 (16.12) | 17.5 (15.21) |
| Number of alcoholic drinks purchased (including additional drinks from study categories only) | 9.06 (9.52) | 9.61 (11.61) | 7.23 (7.78) |
| Number of alcoholic drinks purchased (including all additional drinks) | 9.06 (9.52) | 9.61 (11.61) | 7.48 (7.86) |
| Number of non-alcoholic drinks purchased (including additional drinks from study categories only) | 4.21 (10.76) | 4.56 (6.0) | 7.49 (10.04) |
| Number of non-alcoholic drinks purchased (including all additional drinks) | 6.23 (11.96) | 7.72 (9.87) | 10.02 (13.52) |

**Table G. Full model results for additional outcomes: model estimates (95%confidence interval), p values, percentage changes (95% confidence interval)**

|  | **Model used** | **Reference group: 25% non-alcoholic**  **(n = 207)** | | **Reference group: 50% non-alcoholic**  **(n = 194)** |
| --- | --- | --- | --- | --- |
|  |  | **50% non-alcoholic**  **(n = 194)** | **75% non-alcoholic**  **(n = 207)** | **75% non-alcoholic**  **(n = 207)*** |
| **Additional outcomes (selection):** | | | | |
| Total number of drinks selected | Negative-binomial regression  (weekly units purchased included as covariate) | -0.01 (95%CI -0.20, 0.18)  p = 0.920  -1%  (95%CI -18%, 19%) | -0.04 (95%CI -0.22, 0.14)  p = 0.664  -4%  (95%CI -20%, 15%) | -0.03 (95%CI -0.22, 0.15)  p = 0.743  -3%  (95%CI -20%, 17%) |
| **Additional outcomes (purchasing):** | | | | |
| Total number of drinks purchased (including additional drinks from study categories only) | Negative-binomial regression | 0.08 (95%CI -0.11, 0.27)  p = 0.429  8%  (95%CI -11%, 31%) | 0.133 (95%CI -0.1, 0.33)  p = 0.175  14%  (95%CI -6%, 38%) | 0.06 (95%CI -0.14, 0.25)  p = 0.568  6%  (95%CI -13%, 28%) |
| Total number of drinks purchased (including all additional drinks) | Negative-binomial regression | 0.14 (95%CI -0.05, 0.33)  p = 0.141  15%  (95%CI -5%, 40%) | 0.15,= (95%CI -0.04, 0.35)  p = 0.118  17%  (95%CI -4%, 41%) | 0.01 (95%CI -0.18, 0.20)  p = 0.918  10%  (95%CI -17%, 23%) |
| Number of alcoholic drinks purchased (including additional drinks from study categories only) | Negative-binomial regression | 0.10 (95%CI -0.13, 0.33)  p = 0.409  10%  (95%CI -12%, 39%) | -0.20 (95%CI -0.43, 0.04)  p = 0.103  -18%  (95%CI -35%, 4%) | -0.29 (95%CI -0.52, -0.06)  p = 0.015  -25%  (95%CI -41%, -6%) |
| Number of alcoholic drinks purchased (including all additional drinks) | Negative-binomial regression | 0.10 (95%CI -0.13, 0.32)  p = 0.402  10%  (95%CI -12%, 38%) | -0.17 (95%CI -0.40, 0.06)  p = 0.141  -16%  (95%CI -32%, 6%) | -0.27 (95%CI -0.50, -0.04)  p = 0.022  -24%  (95%CI -39%, -4%) |
| Number of non-alcoholic drinks purchased (including additional drinks from study categories only) | Negative-binomial regression | 0.07 (95%CI -0.33, 0.47)  p = 0.734  7%  (95%CI -28%, 60%) | 0.57 (95%CI 0.17, 0.98_  p = 0.005  78%  (95%CI 18%, 166%) | 0.50 (95%CI 0.10, 0.91)  p = 0.014  66%  (95%CI 10%, 149%) |
| Number of non-alcoholic drinks purchased (including all additional drinks) | Negative-binomial regression | 0.23 (95%CI -0.14, 0.59)  p = 0.223  25%  (95%CI -13%, 80%) | 0.47 (95%CI 0.10, 0.84)  p = 0.011  60%  (95%CI 11%, 132%) | 0.25 (95%CI -0.12, 0.62)  p = 0.187  28%  (95%CI -11%, 85%) |

^1^Note significance threshold is 0.0167 for a 5% alpha

**References**

1. Reynolds JP, Ventsel M, Kosīte D, et al. Impact of decreasing the proportion of higher energy foods and reducing portion sizes on food purchased in worksite cafeterias: A stepped-wedge randomised controlled trial. *PLOS Med* 2021; **18:** e1003743.

1. Options based on representing range of brands and drink types categorised as ‘Premium drinks & mixers’ at Tesco.com, which include: Sparkling water (San Pellegrino); Premium soft drinks - glass bottles (Fentimans, Fever Tree, Bottle Green, Belvoir, Appletiser, Shloer, J2O, Tesco, London Essence) or cans (San Pellegrino, Appletiser, J2O); Fruit blends (J2O); Plastic bottled drinks (Lipton Ice Tea, Oasis); Soda water (Tesco, Schweppes); Tonic water (Fever Tree, Tesco, Schwepps, London Essence); Ginger beer (Fever Tree, Tesco, Fentimans, Old Jamaica); Ginger ale (Schweppes, Tesco).

   *Note*. Fruit juices (cranberry and tomato) and cordials in this Tesco.com category have been excluded due to overlap with drinks aimed at both children and adults. [↑](#footnote-ref-1)
