# Supplementary material for: Impact on alcohol selection and online purchasing of changing the proportion of available non-alcoholic versus alcoholic drinks: A randomised controlled trial
Source: PLoS Med. 2023 Mar 30;20(3):e1004193. doi: 10.1371/journal.pmed.1004193 (PMC10062674; doi:10.1371/journal.pmed.1004193)
Supplement: S1 Study protocol — (DOCX) [file pmed.1004193.s002.docx]

**STUDY PROTOCOL**

**Impact of altering the relative availability of non-alcoholic vs alcoholic drinks on selection and purchasing: online experimental study**

**Anna Blackwell, Natasha Clarke, Jennifer Ferrar, Katie De-Loyde, Mark Pilling, Marcus Munaf****ò, Theresa Marteau, Gareth Hollands**

# Abstract

## Background

Excessive consumption of alcohol increases the risk of a range of diseases, including liver disease and many cancers. One promising intervention to reduce alcohol selection and consumption is increasing the availability of non-alcoholic drinks, relative to the proportion of alcoholic drinks, which has been shown to reduce alcohol selection in online settings. However, there is an absence of evidence concerning the impact of availability on alcohol purchasing. The aim of this study is to estimate the impact of altering the relative availability of a subset of drink options by increasing the proportion of non-alcoholic drinks and decreasing the proportion of alcoholic drinks, on online selection and purchasing of alcohol.

## Methods

*Study design:* Participants will be randomly assigned to one of three conditions in an experiment with a between-subjects factor of drink subset availability (i. 75% non-alcoholic, 25% alcoholic ii. 50% non-alcoholic, 50% alcoholic iii. 25% non-alcoholic, 75% alcoholic), in which the relative proportion presented (%) of alcohol-free beer, alcohol-free cider, alcohol-free wine and soft drinks will vary, compared to alcoholic beer, cider and wine options.

*Primary outcome*: number of alcohol units (i.e. the quantity of pure alcohol) selected (with intention to purchase).

*Procedure*: A sample of adults in England and Wales who consume alcohol (i.e., beer, cider and/or wine) at least weekly, and purchase these drinks at least monthly online at Tesco (minimum £20 monthly spend), will be recruited by a research agency (Roots Research). Participants will be directed to the drink selection task to choose drinks they would like to purchase as part of their next grocery shop. They will then be directed to complete their grocery shop via Tesco online, including the drinks previously selected as well as any other grocery items. They will be asked to confirm their delivery slot within 48 hours of the selection task and send proof of purchase (i.e., itemised receipt) within 48 hours of the delivery date and time.

## Study context

The results of this study will provide information about the impact of altering the relative availability of a subset of drink options, by increasing the presented proportion of non-alcoholic drinks and decreasing the proportion of alcoholic drinks, on the number of alcohol units selected and purchased online. The results will inform future field studies that aim to develop evidence regarding alcohol availability interventions.

Corresponding author: Natasha Clarke, ncc42@medschl.cam.ac.uk

# Background

Excessive alcohol consumption is one of four modifiable behaviours, as well as tobacco use, physical inactivity and unhealthy diet, which are major contributors to non-communicable disease, such as cancer, heart disease and stroke (World Health Organisation, 2018). Traditional approaches to reducing alcohol consumption often involve providing information about harms linked to consumption. Interventions focused on information provision have limited population level effects and may serve to exacerbate health inequalities (Marteau, Hollands, & Fletcher, 2012). Given the known impact of environmental cues on consumption behaviours, an alternative approach is to alter the small-scale environments in which they occur (Hollands et al., 2017).

One promising intervention is increasing the availability of non-alcoholic drinks relative to the proportion of alcoholic drinks, which has been shown to reduce alcohol selection in online settings. According to the Typology of Interventions in Proximal Physical Micro‐Environments (TIPPME) (Hollands et al., 2017), this is classified as an *‘Availability x Product’* intervention. A recent conceptual framework of availability interventions described three possible categories of changes that could be implemented (R. Pechey, Hollands, Carter, & Marteau, 2020). An intervention may: i) alter the absolute availability of options (e.g., increase the total number of non-alcoholic and alcoholic drink items available without changing their relative proportions); ii) alter the relative availability of a subset of options (e.g., increase the proportion of non-alcoholic drinks relative to alcoholic drinks while keeping the overall number of drink items constant; thereby reducing the number of the latter); or iii) alter both the absolute and relative availability (e.g., increase the total number of non-alcoholic drinks, increasing their relative proportion to alcoholic drinks, as well as the total number of drink items).

Research has generally focused on interventions that alter relative availability only, as space restrictions often fix the total number of items within a selection (e.g., in vending machines or cafeterias). However*,* a recent online study found that increasing both the absolute and relative availability, or only the relative availability, of non-alcoholic compared to alcoholic drinks increased their selection (Blackwell et al., 2020). The proportion of participants choosing a non-alcoholic drink increased from 36% (range comprised two non-alcoholic and two alcoholic drinks) or 39% (range comprised four non-alcoholic and four alcoholic drinks) when equal proportions of drink types were available, to 49% when three-quarters of drinks available were non-alcoholic (range comprised six non-alcoholic and two alcoholic drinks). It is notable that there was no meaningful difference in non-alcoholic selection between the two conditions in which there were equal proportions of drink types (i.e., an increase in absolute availability only), which suggests that altering the relative availability (i.e., increasing the proportion of one subset thereby reducing another) may be particularly important for changing selection behaviour.

The current market for alcohol-free beer, alcohol-free wine and alcohol-free spirits is small but rapidly increasing (The Morning Advertiser, 2019). Interventions that increase the availability of these products have the potential to raise consumer awareness through greater exposure, widen consumer choice and the chances of identifying a preferred option, and shift social norms regarding their selection and consumption (R. Pechey et al., 2020). Similar interventions that have increased the proportion of healthier food products demonstrated increases in healthier food purchasing or consumption (Allan, Querstret, Banas, & de Bruin, 2017; Grech & Allman-Farinelli, 2015; Hollands et al., 2019; Rachel Pechey et al., 2019). However, the evidence base for availability interventions regarding alcohol products is small, and there is currently an absence of evidence for the impact on alcohol purchasing or consumption (Hollands et al., 2019).

The aim of this study is to estimate the impact of altering the relative availability of a subset of drink selection options, to increase the proportion of non-alcoholic drinks (alcohol-free beer, cider and wine and soft drinks) and decreasing the proportion of alcoholic drinks (beer, cider and wine) (i.e., decreasing the number of alcohol units within the range of available products), on the number of alcohol units that are i) selected and ii) purchased online.

# Study objective

The primary objective of this study is to estimate the impact on the amount of alcohol units of drinks selected by altering the relative availability of a subset of drink options, by increasing the proportion of non-alcoholic drinks (alcohol-free beer, cider and wine and soft drinks) and decreasing the proportion of alcoholic drinks (beer, cider and wine) presented.

For practical reasons, non-alcoholic drinks of interest will be restricted to products categorised as either alcohol-free beer (≤0.5%^[[1]](#footnote-1)^ alcohol by volume, ABV), alcohol-free cider (≤0.5% ABV), alcohol-free wine (≤0.5% ABV), or soft drinks that are not aimed at children (e.g., premium still or sparkling fruit flavoured drinks, or mixers such as tonic water, soda water or ginger ale). This definition aims to avoid including drinks purchased as part of the grocery shop that may not be considered as alternatives for alcohol or are primarily drinks for children (e.g., tea, coffee, squash, milk-based drinks, juice).

# Hypothesis

Altering the relative availability of a subset of drink options by increasing the proportion of non-alcoholic drinks (alcohol-free beer, cider and wine and soft drinks) and decreasing the proportion of alcoholic drinks (beer, cider and wine) will reduce the number of alcohol units selected.

# Methods

**Study design**

Participants will be randomly assigned to one of three conditions in an experiment with a between-subjects factor of drink subset availability (i. 75% non-alcoholic, 25% alcoholic ii. 50% non-alcoholic, 50% alcoholic iii. 25% non-alcoholic, 75% alcoholic), in which the relative proportion (%) of alcohol-free beer, cider and wine and soft drinks will vary, compared to alcoholic beer, cider and wine options.

## Study site

The studies will be conducted online, designed and hosted on the Qualtrics online survey platform (<http://www.qualtrics.com/>).

## Participants and recruitment

UK adults (N=600 recruited to achieve 510 completers) who consume alcohol (i.e., beer, cider and/or wine) at least weekly, and purchase these drinks at least monthly online from Tesco (minimum £20 monthly spend on these drinks), will be recruited to the study through a recruitment agency (Roots Research: <https://rootsresearch.co.uk/>).

Participants will be reimbursed £25 on study completion. Participants who begin the study but do not complete it will not be reimbursed, which will be explained at the start of the study.

### *Inclusion criteria*

1. Aged 18 years or over;
2. Current Tesco online customer;
3. Regularly consume alcohol – beer, cider and/or wine (i.e., at least once a week);
4. Regularly purchase alcohol – beer, cider and/or wine – online at Tesco (i.e., at least once a month, minimum £20 monthly spend on these drinks);
5. Willing to select drinks from 64 options, shown in an online task, to purchase in next online shop at Tesco;

*(Note: Options will include different brands of beer, cider, wine, alcohol-free beer, alcohol-free cider, alcohol-free wine and soft drinks available at Tesco; no additional drinks from these categories should be added to the Tesco shop)*

1. Willing to complete online shop at Tesco, book a delivery (or Click and Collect) slot, and send the details to the research team within 48 hours of the drink selection task;
2. Willing to send proof of purchase (i.e., itemised receipt) to the research team within 48 hours of delivery or collection date;
3. Have a Tesco delivery or collection address in England or Wales.

### *Sample size determination*

A previous online study compared the impact on drink selection of altering non-alcoholic vs alcoholic drink availability (Blackwell et al., 2020). The results showed that availability of non-alcoholic drink options affected drink selection (binary outcome of alcoholic drink selected, yes/no) in the online setting. The proportion of participants selecting an alcoholic drink decreased from 74% when non-alcoholic drink availability was low (25% of drink options), to 61% when availability was medium (50% of drink options), and 51% when availability was high (75% of drink options) (i.e., a difference of 13% and 10%, respectively, between adjacent groups). However, only a single drink was selected in this online study and there was no intention to purchase the selected drinks, or opportunity to do so. There is limited evidence available within the literature from which to estimate the effect of the intervention on selection behaviour of multiple drink options with the intention to purchase where the outcome is units of alcohol.

A maximum sample size of 600 is possible with available resources. Assuming a sample size of 600 is recruited and allowing for attrition of 15%, this would result in 510 participants (170/group) in the analysis dataset. Based on the primary outcome of the amount of alcoholic units selected, for a two group t-test with alpha of 5% and power 80% an effect size of 0.3 could be detected. Using data from the test run (~5/group) the conservative estimate of the SD is 12.1 (i.e. the maximum group variance observed). This suggests a difference of at least 3.7 units alcohol selected between groups may be detected by a linear regression model.

### *Withdrawal of participants*

Participants will be informed at the start of the study that they are free to withdraw at any time, by closing their web browser before submitting their responses in the online selection task, or not sending subsequent proof of purchase to the study team. Participants will only be reimbursed after the research team receive proof of purchase. If this is not received within seven days of the given delivery or collection date (following two email reminders), the research team will assume that the participant has withdrawn, and they will not be reimbursed. Participants will be made aware of these details in the information sheet before consenting to take part in the study.

## Randomisation

The study will be delivered on the Qualtrics platform, which will use an algorithm to randomise participants into one of the three conditions.

# Intervention

## Availability of non-alcoholic and alcoholic drinks

Participants will view: i. a range of beers, ciders and soft drinks, and ii. a range of wines and soft drinks. These two drink ranges will each show 32 drink options (Table 1). The full list of possible drinks displayed across conditions can be found in Appendix A. The order in which the two drink ranges (beer, cider and soft drinks first or wine and soft drinks first) are presented will be randomised. In each range, the drink options within the three sub-categories (i.e., i. beer and cider, alcohol-free beer and alcohol-free cider, or soft drinks, ii. wine, alcohol-free wine, or soft drinks) will be presented together under their respective headings but their order of presentation will be randomised. The relative availability (proportion) of non-alcoholic (alcohol-free beer, cider or wine alternatives and soft drinks) and alcoholic (beer, cider or wine) drink options – and therefore alcohol units – will vary according to the three conditions. Alcohol-free beer, cider and wine will be clearly labelled to ensure that they are not confused with alcoholic drinks. Each drink option presented will be a different brand, i.e., as the relative availability of non-alcoholic drink options increases, there will be a larger choice of brands from which to choose. Drink images will all be shown as bottles or cans, either as single items or multipacks, depending on the availability of products at Tesco. Participants will be able to select as many drinks as they would like to purchase in their household grocery shop.

# Measures

**Primary outcome measure**

- Number of alcohol units selected (with an intention to purchase). *Participants will be aware that they will be required to purchase the drink items chosen in the selection task and send proof of this to the research team. Therefore, the primary outcome of selection will be made in the context of intention to purchase and provide evidence of this behaviour.*

Units of alcohol – a measure of pure alcohol in a drink with one unit being 10ml or 8g of pure alcohol - will be calculated for all drinks that are >0% ABV (i.e. alcoholic and alcohol-free drinks).

## Secondary outcome measures

- Number of alcoholic drinks selected
- Number of non-alcoholic drinks selected
- Total number of drinks selected
- Proportion of total drinks selected that are alcoholic
- Number of alcohol units purchased
- Number of alcoholic drinks purchased
- Number of non-alcoholic drinks purchased
- Total number of drinks purchased
- Proportion of total drinks purchased that are alcoholic

**Table 1 Relative availability of non-alcoholic and alcoholic drinks displayed in the selection task**

|  | *Drink subset availability* | | |
| --- | --- | --- | --- |
| *Drink range* | *75% non-alcoholic, 25% alcoholic* | *50% non-alcoholic, 50% alcoholic* | *25% non-alcoholic, 75% alcoholic* |
| Beer, cider and soft drinks (n) | *Alcohol-free (AF) beer and cider (12)*  AF lager (4)  AF ale (4)  AF cider (4)  *Soft drinks (12)*  *Beer and cider (8)*  Lager (3)  Ale (3)  Cider (2) | *AF beer and cider (8)*  AF lager (3)  AF ale (3)  AF cider (2)  *Soft drinks (8)*  *Beer and cider (16)*  Lager (6)  Ale (6)  Cider (4) | *AF beer and cider (4)*  AF lager (2)  AF ale (1)  AF cider (1)  *Soft drinks (4)*    *Beer and cider (24)*  Lager (10)  Ale (8)  Cider (6) |
| Wine and soft drinks (n) | *AF wine (12)*  AF red wine (3)  AF white wine (5)  AF rose or sparkling wine (4)  *Soft drinks (12)*    *Wine (8)*  Red wine (3)  White wine (3)  Rose or sparkling wine (2) | *AF wine (8)*  AF red wine (3)  AF white wine (3)  AF rose or sparkling wine (2)  *Soft drinks (8)*    W*ine (16)*  Red wine (6)  White wine (6)  Rose or sparkling wine (4) | *AF wine (4)*  AF red wine (1)  AF white wine (1)  AF rose or sparkling wine (2)  *Soft drinks (4)*    W*ine (24)*  Red wine (9)  White wine (9)  Rose or sparkling wine (6) |

The drink type, brand, volume of container(s) (ml), alcohol strength (ABV), and price (£) of each drink selected and purchased will be recorded. The number of drinks will be recorded *per individual drink container* (i.e., a 4x330ml beer multipack will be recorded as four drink items).

Price promotions and variations for all drinks included in the selection task will be checked every month via Tesco.com and recorded. Prices shown in the task will reflect the full price on Tesco.com throughout the study.

## Additional measures

*Demographics*

Age, gender, and highest qualification attained (with the options: ‘Higher Education or professional / vocational equivalents’, ‘A levels or vocational level 3 or equivalents’, ‘GCSE / O Level grade A*‐C or vocational level 2 or equivalents’, ‘Qualifications at level 1 and below’, ‘Other qualifications: level unknown’, or ‘No qualifications’) (ONS, 2015).

*Household members*

Participants will be asked about the members of their household, including the number of adults (aged 18+) in their household who have been included for consideration in the drink selection and the number of children (aged <18) in the household.

*Drinking behaviour risk*

The Alcohol Use Disorders Identification Test (AUDIT) (Bohn, Babor, & Kranzler, 1995) questions will be used to assess the level of risk associated with participants’ drinking behaviour.

*Weekly unit consumption*

Participants will be asked to enter the number of drinks they have consumed and purchased over the previous seven days, which will be used to calculate the number of alcohol units.

*Open text comment*

Participants will be asked to provide comments on task, such as explaining their choice of drinks, via an open text box to inform future studies in this area.

**Refinement of study procedure**

We conducted a small test run of the study procedures (N=14) to identify any issues and collect feedback from participants, which was used to further develop and refine the study procedure and instructions, prior to the full study. The following measures were recorded but these test run data will not be included as part of the final dataset:

- Number of alcoholic units selected
- Number of alcoholic and non-alcoholic drinks selected and purchased
- Percentage exact matches, close matches (i.e., like-for-like swaps, based on drink type: such as alcoholic red wine, non-alcoholic beer), and non-matches between selection and purchasing
- Dropout rate from selection to purchasing task
- Average time to receive confirmation of delivery or collection slot and proof of purchase
- Average time to extract information from receipts
- Open ended feedback on participants’ experience completing the tasks

# Procedure

Participants will be recruited by a research agency (Roots Research: <https://rootsresearch.co.uk/>). They will be provided with a participant information sheet, instruction guide and a link to the study on the Qualtrics platform. The information sheet will explain the study and what they will be required to do. It will be made clear that they will be asked to purchase only the drinks that they select in the task (or like-for-like alternatives if they are out of stock) in their next grocery shop, and that the options may not include all of their usual brands. Before commencing the online study, participants will be presented with task instructions, as well as links to the participant information sheet and instruction guide for reference. They will then complete a tick-box consent page and be directed to the drink selection task.

Participants will be randomised to one of three drink subset availability conditions (Table 1). They will be shown two drink ranges of 32 options (64 drink items in total): i. beer, cider or soft drinks, and ii. wine or soft drinks, and asked to choose all the drinks they would like to purchase as part of their next online grocery shop at Tesco. Participants will be shown their total drink selection and price, and they will be given an opportunity to go back and amend their selection before continuing with the study. Then participants will be asked to provide their email address (to be entered twice to ensure accuracy) and answer questions regarding their demographics and typical drinking behaviour, and to briefly explain their drink choices. After completing the selection task, participants will be automatically sent an email detailing their selected drinks.

When participants complete the selection task, they will be prompted to check their email and provided with instructions to complete the purchasing stage of the study, alongside a link to the Tesco website. The instructions will also be included in the automatic email they are sent and will request that participants place all of their selected drinks in their online Tesco shopping basket, along with any other grocery items, book their delivery or collection slot and confirm their delivery or collection time and date within 48 hours, via a survey link in the email. Participants will be sent an email on the day of their delivery or collection with a request to send proof of purchase (i.e., a copy of their itemised receipt) to the research team within 48 hours. Up to two follow-up email reminders will be sent, two and four days after this time has lapsed. Researchers will record the drink information from the itemised receipts. Participants will be debriefed, including information about how they can find out more about the study and contact details for the research team, and reimbursed for their time participating in the study.

# Statistical plan

Analysis will be conducted in SPSS (version 24 or later) or similar packages.

A CONSORT flow chart will be produced to indicate the numbers approached, recruited and randomised to the various study arms.

## Primary outcome: Selection

A generalised linear model or similar - will be used to analyse the primary outcome (the number of alcohol units selected) between the three study arms. The reference group will be the 25% non-alcoholic, 75% alcoholic group, which is most representative of real world settings in which alcoholic drinks are more predominant than non-alcoholic drinks. Adjustment will be made for whether beer or wine was shown to the participant first (as a dichotomous random factor).

## Secondary outcomes

The above analysis will be repeated for most of the secondary outcomes. For the proportion of total drinks selected and purchased that are alcoholic we will use a binary logistic model based on the counts of drinks.

Each pairwise effect will be reported as a difference in means with 95% confidence interval (CI) of the mean, t statistics and p-values. For these pairwise comparisons only, a significant p value of 5%/3 will be adopted due to multiple comparisons. An umbrella p value and F statistic, between all three study arms, will also be reported.

## Tertiary analysis

*Per-protocol analyses: discrepancy between outcomes for selection and purchasing*

The threshold for, and definition of, exact or close product matches between selected and purchased products will be established when developing the detailed statistical analysis plan, which will be blinded to any potential effects of using different thresholds on study results and registered prior to conducting the statistical analysis. Participants whose purchased products fall below an agreed threshold for matches with selected products will be excluded for per-protocol analyses. Provisionally, we anticipate that this will consist of two analyses using differently stringent definitions of close matching between selected and purchased items: First, a stringent analysis that requires exact matching of selected and purchased items irrespective of any justification for these differing, and second, a less stringent analysis where differences between selected and purchased items are accepted providing participants give an explicit justification for any differences e.g. items unavailable for purchase.

# Research governance

In the UK, research will adhere to the [Wellcome Trust Policy on Good Research Practice](https://wellcome.org/grant-funding/guidance/good-research-practice-guidelines) and the [UK Policy Framework for Health and Social Care Research](https://www.medschl.cam.ac.uk/wp-content/uploads/2014/02/uk-policy-framework-health-social-care-research.pdf). Researchers also follow the principles laid out in the [UK concordat to support research integrity](https://www.universitiesuk.ac.uk/_layouts/15/UUK.Internet2013/UserManagement/uuklogin.aspx?ReturnUrl=%2fpolicy-and-analysis%2freports%2f_layouts%2f15%2fAuthenticate.aspx%3fSource%3d%252Fpolicy%252Dand%252Danalysis%252Freports%252FPages%252Fresearch%252Dconcordat%252Easpx&Source=%2Fpolicy%2Dand%2Danalysis%2Freports%2FPages%2Fresearch%2Dconcordat%2Easpx#.UvOwgEJ_vnA).

## Ethical considerations and informed consent

Ethics approval has been obtained from the School of Psychological Science Research Ethics Committee at the University of Bristol (reference: 116124). Participants will receive information at the start of the survey. The study will be closed online once the required number of participants have been recruited. Participants will be given sufficient time to read the information, consider any implications, and raise any questions with the investigators prior to deciding to participate. Consent will then be obtained. Participants will be informed that they are free to withdraw at any time (see above).

## Sponsorship

The University of Bristol will sponsor this study.

## Safety

As this is an online experiment, we do not foresee any risks to participants.

The University of Bristol holds appropriate liability insurance for research studies involving human participants. If required further information can be found here: <http://www.bristol.ac.uk/secretary/insurance/liability-insurance/>

Indemnity is provided to the University of Cambridge and its employees for legal liability to pay damages for injury caused to volunteers participating in the study under Public Liability/Excess of Loss Public Liability, and Professional Indemnity insurance policies.

## Data management

All aspects of the General Data Protection Regulation, Data Protection Act 2018 and the Freedom of Information Act 2000 will be adhered to. All personal data will be treated as confidential.

*Participant Identifiable Data (PID)*

The study team at the University of Bristol will use participant email addresses to contact them during the study (i.e., an automated email will be generated in the Qualtrics platform to send participants a record of their drink selection). Participants will be asked to email proof of purchase to the study team. Email address and receipts will be stored in password protected files and will be destroyed after data collection is complete. All personally identifiable information collected will be stored securely by the study team at the University of Bristol and will be kept confidential. The data may only be accessed via a secure website which requires log-in credentials. Only study personnel will have access to these data.

### *Anonymised study data*

All study data will be anonymised using a unique numeric identifier. Study data will be stored on an encrypted cloud server after completion. The data may only be accessed via a secure website which requires log-in credentials. Only study personnel will have access to these data.

### *Data sharing*

Anonymous study data may be shared with collaborators for the purposes of analysis and results interpretation under appropriate collaboration agreements.

### *Long-term data archiving*

At the end of the study, electronic study data (including finalised data sheet) will be transferred to a designated University of Bristol Research Data Storage Facility for long-term archiving. Study data will be kept for a minimum of 20 years.

### *Open data*

At the appropriate time the data sheet will be locked and made open using the

University of Bristol Research Data Repository and / or Open Science Framework.

### *Revoked data*

If a participant decides that they do not want their data used after their participation, they can request that the data are withdrawn. They can request up to the point that data collection is complete and made anonymous, after which, it will not be possible to identify their data for removal. Participants will be made aware of prior to consenting to take part in the study.

## Quality control and quality assurance

The investigators will be responsible for data quality. Data input from participants receipts and compared to drinks chosen in the selection task will undergo a reliability check (20% check by independent researcher). If an error rate greater than 1% is obtained the data will be re-inputted in full and assessed again.

# Publication policy

The findings from this research study may be published in an appropriate scientific journal (and made available open access), and/or presented at an appropriate meeting. Study data will be collected and held by the study investigators. The data will be made available for sharing via a University of Bristol online data repository and / or Open Science Framework.

# Study Personnel

Anna K M Blackwell

School of Psychological Science

University of Bristol

12a Priory Rd

Bristol, BS8 1TU

Email: [anna.blackwell@bristol.ac.uk](mailto:anna.blackwell@bristol.ac.uk)

Natasha Clarke

Behaviour and Health Research Unit

University of Cambridge

Institute of Public Health

Cambridge CB2 0SR

Email: [ncc42@medschl.cam.ac.uk](mailto:ncc42@medschl.cam.ac.uk)

Jennifer Ferrar

School of Psychological Science

University of Bristol

12a Priory Rd

Bristol, BS8 1TU

Email: [jennifer.ferrar@bristol.ac.uk](mailto:jennifer.ferrar@bristol.ac.uk)

Katie De-loyde

School of Psychological Science

University of Bristol

12a Priory Rd

Bristol, BS8 1TU

Email: [kd16662@bristol.ac.uk](mailto:kd16662@bristol.ac.uk)

Mark Pilling

Behaviour and Health Research Unit

University of Cambridge

Institute of Public Health

Cambridge CB2 0SR

Email: [mark.pilling@medschl.cam.ac.uk](mailto:mark.pilling@medschl.cam.ac.uk)

Marcus R Munafò

School of Psychological Science

University of Bristol

12a Priory Rd

Bristol, BS8 1TU

Email: [marcus.munafo@bristol.ac.uk](mailto:marcus.munafo@bristol.ac.uk)

Theresa M Marteau

Behaviour and Health Research Unit

University of Cambridge

Institute of Public Health

Cambridge CB2 0SR

Email: [tm388@cam.ac.uk](mailto:tm388@cam.ac.uk)

Gareth J Hollands

Behaviour and Health Research Unit

University of Cambridge

Institute of Public Health

Cambridge CB2 0SR

Email: [gareth.hollands@medschl.cam.ac.uk](mailto:gareth.hollands@medschl.cam.ac.uk)

# Funding source

This work is supported by a Collaborative Award in Science from the Wellcome Trust (Behaviour Change by Design: 206853/Z/17/Z) awarded to Theresa Marteau, Paul Fletcher, Gareth Hollands and Marcus Munafò. The funder is not involved in the study design or data analysis.

# Conflicts of interest

The study investigators have no known conflicts of interest to declare.

**References**

Allan, J., Querstret, D., Banas, K., & de Bruin, M. (2017). Environmental interventions for altering eating behaviours of employees in the workplace: a systematic review. *18*(2), 214-226. doi:10.1111/obr.12470

Blackwell, A. K. M., De-loyde, K., Hollands, G. J., Morris, R. W., Brocklebank, L. A., Maynard, O. M., . . . Munafò, M. R. (2020). The impact on selection of non-alcoholic vs alcoholic drink availability: an online experiment. *BMC Public Health, 20*, 526.

Grech, A., & Allman-Farinelli, M. (2015). A systematic literature review of nutrition interventions in vending machines that encourage consumers to make healthier choices. *Obesity Reviews, 16*(12), 1030-1041. doi:doi:10.1111/obr.12311

Hollands, G. J., Bignardi, G., Johnston, M., Kelly, M. P., Ogilvie, D., Petticrew, M., . . . Marteau, T. M. (2017). The TIPPME intervention typology for changing environments to change behaviour. *Nature Human Behaviour, 1*, 0140. doi:10.1038/s41562-017-0140

<https://www.nature.com/articles/s41562-017-0140#supplementary-information>

Hollands, G. J., Carter, P., Anwer, S., King, S. E., Jebb, S. A., Ogilvie, D., . . . Marteau, T. M. (2019). Altering the availability or proximity of food, alcohol, and tobacco products to change their selection and consumption. *Cochrane Database of Systematic Reviews*(9), CD012573. doi:10.1002/14651858.CD012573.pub3

Marteau, T. M., Hollands, G. J., & Fletcher, P. C. (2012). Changing Human Behavior to Prevent Disease: The Importance of Targeting Automatic Processes. *Science, 337*(6101), 1492. doi:10.1126/science.1226918

ONS. (2015). Harmonised Concepts and Questions for Social Data Sources. Primary Principles: Other Primary Principles (Version 5.4). Retrieved from <http://www.ons.gov.uk/ons/guide-method/harmonisation/primary-set-of-harmonised-concepts-and-questions/index.html>

Pechey, R., Cartwright, E., Pilling, M., Hollands, G. J., Vasiljevic, M., Jebb, S. A., & Marteau, T. M. (2019). Impact of increasing the proportion of healthier foods available on energy purchased in worksite cafeterias: A stepped wedge randomized controlled pilot trial. *Appetite, 133*, 286-296. doi:<https://doi.org/10.1016/j.appet.2018.11.013>

Pechey, R., Hollands, G. J., Carter, P., & Marteau, T. M. (2020). Altering the availability of products within physical micro-environments: A conceptual framework. *BMC Public Health, 20*(01/10/2019), 986.

The Morning Advertiser. (2019). The boom of alcohol-free is a sticking trend. Retrieved from <https://www.morningadvertiser.co.uk/Article/2019/05/20/How-much-has-the-no-alcohol-category-grown>

World Health Organisation. (2018). Global status report on alcohol and health 2018. Retrieved from <https://www.who.int/substance_abuse/publications/global_alcohol_report/en/>

# Appendix A

Alcohol-free beer, alcohol-free cider and alcohol-free wine options used in the task (Table 2) were selected based on brand and size matches, where possible, with alcohol options available online at Tesco.com. Additional alcoholic beer, cider and wine was selected based on the leading brands of lager, ale, mild and stout^[[2]](#footnote-2)^, cider^[[3]](#footnote-3)^ and wine^[[4]](#footnote-4)^ in Great Britain according to the number of users. Products shown in bold are those used in the ‘50% non-alcoholic, 50% alcoholic’ condition.

Table 2 Drink options used in the selection task (Prices based on Tesco.com)

| ***Brand name*** | ***ABV*** | ***Volume*** | ***Price*** | ***Alcohol / alcohol-free match*** |
| --- | --- | --- | --- | --- |
| ***Alcohol-free Beer*** | | | | |
| **Heineken Alcohol Free Beer 12 X 330Ml** | **0.00%** | **12x330ml** | **£8.00 (£2.03/l)** | **Y** |
| **Peroni Liberia Alcohol Free 4X330ml Bottle** | **0.00%** | **4x330ml** | **£4.50 (£3.41/l)** | **Y** |
| **San Miguel 0.0% Alcohol Free Lager 4X330ml** | **0.00%** | **4x330ml** | **£3.50 (£2.66/l)** | **Y** |
| Becks Blue Alcohol Free 15X275ml | 0.05% | 15x275ml | £7.00 (£1.70/l) | Brand only (+ exposure) |
| **Brewdog Punk Af 4X330ml** | **0.50%** | **4x330ml** | **£4.50 (£3.41/l)** | **Y** |
| **Hoegaarden Wit Blanche Wheat Beer 0.0 4X330ml** | **0.00%** | **4x330ml** | **£4.00 (£3.04/l)** | **Y** |
| **Adnams Ghost Ship Bottle Beer 0.5% 500Ml** | **0.50%** | **500ml** | **£1.30 (£2.60/l)** | **Y** |
| Doom Bar Zero Amber Ale 500Ml | 0.00% | 500ml | £1.30 (£2.60/l) | Y |
| ***Alcohol-free Cider*** | | | | |
| **Friels Low Alcohol Cider 4X330ml** | **0.50%** | **4x330ml** | **£3.50 (£2.66/l);** | **Y** |
| **Kopparberg Premium Cider Mixed Fruit Alcohol Free 4X330ml** | **0.05%** | **4x330ml** | **£3.50 (£2.66/l)** | **Y** |
| Stowford Press Apple Cider Low Alcohol 500Ml | 0.50% | 500ml | £1.30 (£2.60/l) | Brand only |
| Kopparberg Alcohol Free Pear Cider 500Ml | 0.05% | 500ml | £1.30 (£2.60/l) | Y |
| ***Alcohol-free Wine*** | | | | |
| **Lindeman's Alcohol Free Cabernet Sauvignon 750Ml** | **0.50%** | **750ml** | **£4.00** | **Brand only** |
| **Tesco Low Alcohol Cabernet Tempranillo 76Cl** | **0.50%** | **750ml** | **£2.75** | **Y** |
| **Eisberg Merlot Alcohol Free Wine 75Cl** | **0.00%** | **750ml** | **£3.50** | **Grape only** |
| **Hardys Alcohol Free Chardonnay 75Cl** | **0.05%** | **750ml** | **£5.00** | **Y** |
| **Lindeman's Alcohol Free Semillon Chardonnay 750Ml** | **0.50%** | **750ml** | **£4.00** | **Brand only** |
| **Tesco Low Alcohol Sauvignon Blanc 75Cl** | **0.50%** | **750ml** | **£2.75** | **Y** |
| Eisberg Sauvignon 75Cl Alcohol Free | 0.00% | 750ml | £3.50 | Grape only |
| Mcguigan Zero Alcohol Free Sauvignon Blanc 75Cl | 0.05% | 750ml | £5.00 | Brand only |
| **Tesco Low Alcohol Garnacha Rose 75Cl** | **0.50%** | **750ml** | **£2.75** | **Y** |
| Eisberg Rose 75Cl Alcohol Free | 0.00% | 750ml | £3.50 | Type only |
| **Freixenet 0.0% Alcohol Free Sparkling 75Cl** | **0.00%** | **750ml** | **£5.00** | **Y** |
| Tesco Low Alcohol Sparkling White Wine 75Cl | 0.50% | 750ml | £2.75 | Y |
| ***Soft drinks^[[5]](#footnote-5)^ (used in alcohol-free beer, cider and soft drinks selection)*** | | | | |
| **Fentimans Curiosity Cola 4X275ml** | **n/a** | **4x275ml** | **£4.53 (£0.41/100ml)** | **n/a** |
| **San Pellegrino Sparkling Water 6 X 1 Litre** | **n/a** | **6 x 1l** | **£5.50 (£0.09/100ml)** | **n/a** |
| **Belvoir Light Elderflower Presse 750Ml** | **n/a** | **750ml** | **£2.49 (£0.33/100ml)** | **n/a** |
| **San Pellegrino Sparkling Limonata 6X330ml** | **n/a** | **6X330ml** | **£3.79 (£0.19/100ml)** | **n/a** |
| **J20 Orange & Passion Fruit 6 X 275Ml** | **n/a** | **6X275Ml** | **£6.00 (£0.36/100ml)** | **n/a** |
| **Tesco Soda Water 1Litre** | **n/a** | **1l** | **£0.50 (£0.05/100ml)** | **n/a** |
| **Schweppes Tonic Water 12 X 150Ml** | **n/a** | **12 X 150ml** | **£4.00 (£0.22/100ml)** | **n/a** |
| **Fentimans Traditional Ginger Beer 4X275ml** | **n/a** | **4x275ml** | **£4.53 (£0.41/100ml)** | **n/a** |
| Appletiser 100% Sparkling Apple Juice 750Ml | n/a | 750ml | £1.45 (£0.19/100ml) | n/a |
| Lipton Ice Tea Peach Flavour 1.25 Litre Bottle | n/a | 1.25l | £1.60 (£0.13/100ml) | n/a |
| Tesco Low Calorie Indian Tonic Water Cans 6X250ml | n/a | 6X250ml | £2.25 (£0.15/100ml) | n/a |
| Tesco Low Calorie Ginger Ale 1 Litre | n/a | 1l | £0.50 (£0.05/100ml) | n/a |
| ***Soft drinks (used in alcohol-free wine and soft drinks selection)*** | | | | |
| **J2O Spritz Apple Watermelon 6X275ml** | **n/a** | **6X275ml** | **£6.00 (£0.36/100ml)** | **n/a** |
| **Shloer Sparkling White Grape Juice 750Ml** | **n/a** | **750ml** | **£2.20 (£0.29/100ml)** | **n/a** |
| **Fentimans Traditional Rose Lemonade 750Ml** | **n/a** | **750ml** | **£2.95 (£0.39/100ml)** | **n/a** |
| **San Pellegrino Aranciata Rossa 6 Pack Can 330Ml** | **n/a** | **6X330ml** | **£3.79 (£0.19/100ml)** | **n/a** |
| **Oasis Summer Fruit 1.5 Litre Bottle** | **n/a** | **1.5l** | **£1.00 (£0.07/100ml)** | **n/a** |
| **Schweppes Soda Water 1 Litre** | **n/a** | **1l** | **£1.50 (£0.15/100ml)** | **n/a** |
| **London Essence Orange & Elderflower Tonic 6 X 150Ml** | **n/a** | **6X150ml** | **£3.25 (£0.36/100ml)** | **n/a** |
| **Schweppes Canada Dry Ginger Ale 1Ltr** | **n/a** | **1l** | **£1.50 (£0.15/100ml)** | **n/a** |
| Bottlegreen Elderflower Presse 75Cl | n/a | 750ml | £2.49 (£0.33/100ml) | n/a |
| J20 Apple & Raspberry 275Ml X 6 | n/a | 6x275ml | £6.00 (£0.36/100ml) | n/a |
| Fever-Tree Indian Tonic Water 8 X 150Ml | n/a | 8 X 150Ml | £4.25 (£0.36/100ml) | n/a |
| Tesco No Added Sugar Ginger Beer 4 X 330Ml | n/a | 4 X 330Ml | £0.99 (£0.08/100ml) | n/a |
| ***Beer*** | | | | |
| **Heineken 12X330ml Bottles** | **5.00%** | **12x330ml** | **£11.00 (£2.78/l)** | **Y** |
| **Peroni Nastro Azzurro 4X330ml Bottles** | **5.10%** | **4x330ml** | **£6.50 (£4.93/l)** | **Y** |
| **San Miguel 4X330ml** | **5.00%** | **4x330ml** | **£4.50 (£3.41/l)** | **Y** |
| **Becks Lager Beer 20 X 275Ml** | **4.00%** | **20x275ml** | **£10.00 (£1.82/l)** | **Larger multipack** |
| **Budweiser 15 X 440Ml** | **4.50%** | **15x440ml** | **£12.00 (£1.82/l)** | **Addition** |
| **Stella Artois Premium Lager 4 X 568Ml** | **4.60%** | **4x568ml** | **£5.39 (£2.38/l)** | **Addition** |
| Fosters 4X440ml | 4.00% | 4x440ml | £3.49 (£1.99/l) | Addition |
| Carling Lager 18X440ml | 4.00% | 18x440ml | £9.99 (£1.27/l) | Addition |
| Amstel Lager Beer Can 4 X 440Ml | 4.10% | 4x440ml | £4.00 (£2.28/l) | Addition |
| Guinness Draught 4X440ml | 4.10% | 4x440ml | £4.49 (£2.56/l) | Addition |
| **Brewdog Punk Ipa 4X330ml** | **5.40%** | **4x330ml** | **£6.00 (£4.55/l)** | **Y** |
| **Hoegaarden White Beer 4X330 Ml Bottles** | **4.90%** | **4x330ml** | **£4.50 (£3.41/l)** | **Y** |
| **Adnams Ghost Ship 500Ml** | **4.30%** | **500ml** | **£1.70 (£3.40/l)** | **Y** |
| **Sharps Doom Bar 500Ml** | **4.30%** | **500ml** | **£1.49 (£2.98/l)** | **Y** |
| **Hobgoblin Ipa 500Ml** | **5.30%** | **500ml** | **£1.70 (£3.40/l)** | **Addition** |
| **Old Speckled Hen Can 4 X 500Ml** | **5.00%** | **4x500ml** | **£4.29 (£2.15/l)** | **Addition** |
| Abbot Ale Strong Bitter 4X500ml Cans | 5.00% | 4x500ml | £4.49 (£2.25/l) | Addition |
| Fullers London Pride 500Ml | 4.70% | 500ml | £1.70 (£3.40/l) | Addition |
| ***Cider*** | | | | |
| **Friels Vintage Cider 4 X 330Ml** | **7.40%** | **4x330ml** | **£4.50 (£3.41/l)** | **Y** |
| **Kopparberg Mixed Fruit Cider 4X330ml Bottle** | **4.00%** | **4x330ml** | **£5.00 (£3.79/l)** | **Y** |
| **Stowford Press Apple Cider 4 X 440Ml Can** | **4.50%** | **4x440ml** | **£3.50 (£1.99/l)** | **Multipack vs single** |
| **Kopparberg Pear 500Ml Bottle** | **4.50%** | **500ml** | **£2.00 (£4.00/l)** | **Y** |
| Strongbow Original Cider 4X440ml | 4.50% | 4x440ml | £4.00 (£2.28/l) | Addition |
| Bulmers Original Premium Cider 8 X 500Ml | 4.50% | 8x500ml | £6.00 (£1.50/l) | Addition |
| ***Wine*** | | | | |
| **Lindeman's Bin 50 Shiraz 75Cl** | **13.50%** | **750ml** | **£7.00** | **Brand only** |
| **Tesco Spanish Tempranillo 75Cl** | **12.00%** | **750ml** | **£3.69** | **Similar** |
| **Hardys Varietal Range Merlot 75Cl** | **13.00%** | **750ml** | **£6.00** | **Grape only** |
| **Yellow Tail Pinot Noir 75Cl** | **13.50%** | **750ml** | **£7.00** | **Addition** |
| **Gallo Family Vineyards Merlot 75Cl** | **13.00%** | **750ml** | **£6.00** | **Addition** |
| **Wolf Blass Yellow Label Cabernet Sauvignon 75Cl** | **13.50%** | **750ml** | **£8.00** | **Addition** |
| Campo Viejo Rioja Garnacha 75Cl | 14.00% | 750ml | £8.00 | Addition |
| Kumala Reserve Malbec 75Cl | 13.50% | 750ml | £7.00 | Addition |
| Mcguigan Reserve Cabernet 75Cl | 12.00% | 750ml | £7.00 | Addition |
| **Hardys Crest Chardonnay 75Cl** | **13.00%** | **750ml** | **£7.00** | **Y** |
| **Lindeman's Bin 65 Chardonnay 75Cl** | **12.50%** | **750ml** | **£7.00** | **Similar** |
| **Wolf Blass Yellow Label Sauvignon Blanc 75Cl** | **13.00%** | **750ml** | **£8.00** | **Grape only** |
| **Mcguigan Reserve Chardonnay 75Cl** | **12.50%** | **750ml** | **£7.00** | **Brand only** |
| **Tesco Finest Marlborough Sauvignon Blanc 75Cl** | **12.50%** | **750ml** | **£8.00** | **Y** |
| **Yellow Tail Pinot Grigio 75Cl** | **11.50%** | **750ml** | **£7.00** | **Addition** |
| First Cape Special Cuvee Chenin Blanc | 12.50% | 750ml | £6.00 | Addition |
| Isla Negra Sauvignon Blanc Px 75Cl | 12.00% | 750ml | £5.00 | Addition |
| Kumala Reserve Chenin Blanc 75Cl | 13.00% | 750ml | £7.00 | Addition |
| **Tesco Tempranillo Garnacha Rose 75Cl** | **11.50%** | **750ml** | **£4.50** | **Y** |
| **Blossom Hill White Zinfandel 75Cl** | **11.00%** | **750ml** | **£6.00** | **Type only** |
| Yellow Tail Rose 75Cl | 12.00% | 750ml | £7.00 | Addition |
| **Freixenet Prosecco Doc 75Cl** | **11.00%** | **750ml** | **£12.00** | **Y** |
| **Tesco Finest Prosecco Doc 75Cl** | **11.00%** | **750ml** | **£8.00** | **Y** |
| Finest Prosecco Valdobbiadene Docg 75Cl | 11.50% | 750ml | £10.00 | Addition |

1. *Note*: The UK government recommend that the term ‘alcohol-free’ is used for drinks 0.0-0.05%ABV; ‘de-alcoholised’ is used for drinks ≤0.5% ABV where alcohol has been extracted; and ‘low alcohol’ is used for drinks ≤1.2%ABV: <https://www.gov.uk/government/publications/low-alcohol-descriptors>. The present study is using the term ‘alcohol-free’ more inclusively to include drinks ≤0.5% ABV to avoid confusion from using multiple terms. Other drinks containing ≤0.5% ABV, not associated with alcohol, such as ginger beer and kombuchas, can be sold as soft drinks without labelling and ‘alcohol-free’ is an umbrella term more generally recognised to include beer, wine, cider, and spirit alternatives containing ≤0.5% ABV: <https://joinclubsoda.com/frequently-asked-questions/>. [↑](#footnote-ref-1)
2. Statista (2020) Leading brands of beer in Great Britain 2019, by number of users, Statista Research Department, <https://www.statista.com/statistics/868499/leading-brands-of-beer-in-the-uk/> [↑](#footnote-ref-2)
3. Statista (2020) Leading brands of cider in Great Britain 2019, by number of users, Statista Research Department, <https://www.statista.com/statistics/317609/leading-brands-of-cider-in-the-uk/> [↑](#footnote-ref-3)
4. Statista (2020) Leading brands of still wine in Great Britain 2019, by number of users, Statista Research Department, <https://www.statista.com/statistics/304150/leading-brands-of-wine-including-sparkling-gb-in-the-uk/> [↑](#footnote-ref-4)
5. Options based on representing range of brands and drink types categorised as ‘Premium drinks & mixers’ at Tesco.com, which include: Sparkling water (San Pellegrino); Premium soft drinks - glass bottles (Fentimans, Fever Tree, Bottle Green, Belvoir, Appletiser, Shloer, J2O, Tesco, London Essence) or cans (San Pellegrino, Appletiser, J2O); Fruit blends (J2O); Plastic bottled drinks (Lipton Ice Tea, Oasis); Soda water (Tesco, Schwepps); Tonic water (Fever Tree, Tesco, Schwepps, London Essence); Ginger beer (Fever Tree, Tesco, Fentimans, Old Jamaica); Ginger ale (Schwepps, Tesco).

   *Note*. Fruit juices (cranberry and tomato) and cordials in this Tesco.com category have been excluded due to overlap with drinks aimed at both children and adults. [↑](#footnote-ref-5)
