# Supplementary material for: Impact on alcohol selection and online purchasing of changing the proportion of available non-alcoholic versus alcoholic drinks: A randomised controlled trial
Source: PLoS Med. 2023 Mar 30;20(3):e1004193. doi: 10.1371/journal.pmed.1004193 (PMC10062674; doi:10.1371/journal.pmed.1004193)
Supplement: S1 Analysis plan — (DOCX) [file pmed.1004193.s003.docx]

ANALYSIS PLAN

**Impact of altering the relative availability of non-alcoholic vs alcoholic drinks on selection and purchasing: online experimental study**

**Natasha Clarke, Anna Blackwell, Jennifer Ferrar, Katie De-Loyde, Mark Pilling, Marcus Munaf****ò, Theresa Marteau, Gareth Hollands**

## Changes to protocol

No umbrella p value or F statistic will be reported for the secondary outcomes.

We did not randomise the two drink ranges (‘beer, cider and soft drinks’ or ‘wine and soft drinks’) and instead randomised the order of alcoholic and non-alcoholic drink sub-categories in each of the beer and wine categories. We will not include the randomisation order of the drink sub-categories in the analysis due to a lack of data-points per level. We are therefore not including drink randomisation variables in any of the models.

We have added an outcome a and b for each purchasing outcome to include more and less stringent definitions of additional drink categories and separated outcomes into primary, secondary and additional outcomes.

## Study design

Between-subjects design where participants were randomly assigned to one of three differing alcoholic / non-alcoholic drink conditions. The conditions varied by relative proportion (%) of alcohol-free beer, cider and wine and soft drinks varied, compared to alcoholic beer, cider and wine:

1. 75% non-alcoholic, 25% alcoholic
2. 50% non-alcoholic, 50% alcoholic
3. 25% non-alcoholic, 75% alcoholic

# Hypothesis

Altering the relative availability of a subset of drink options by increasing the proportion of non-alcoholic drinks (alcohol-free beer, cider and wine and soft drinks) and decreasing the proportion of alcoholic drinks (beer, cider and wine) will reduce the number of alcohol units selected.

#### Hypothesis testing

Two co-primary hypothesis tests will be conducted, where the reference group will be group 3, and therefore there will be two tests against this group (1 vs. 3 and 2 vs. 3). A 5%/2 adjustment to the significance threshold will be made. A third comparison will also be made between groups 1 vs. 2. This will be a secondary test and a 5%/3 adjustment to the significance threshold will be made for this test.

# Study objective

The primary objective of this study is to estimate the impact on the amount of alcohol units of drinks selected by altering the relative availability of a subset of drink options, by increasing the proportion of non-alcoholic drinks (alcohol-free beer, cider and wine and soft drinks) and decreasing the proportion of alcoholic drinks (beer, cider and wine) presented.

**Outcomes**

#### Primary outcome

Number of alcohol units selected (with an intention to purchase).

**Secondary outcomes**

1. Number of alcoholic drinks selected
2. Number of non-alcoholic drinks selected
3. Number of alcohol units purchased
   1. including additional drinks from study categories only
   2. including all additional drinks
4. Proportion of total drinks selected that are alcoholic
5. Proportion of total drinks purchased that are alcoholic
   1. including additional drinks from study categories only
   2. including all additional drinks

**Additional outcomes**

1. Total number of drinks selected
2. Total number of drinks purchased
3. Number of alcoholic drinks purchased
   1. including all additional drinks
   2. including additional drinks from study categories only
4. Number of non-alcoholic drinks purchased
   1. including additional drinks from study categories only
   2. including all additional drinks

#### Additional measures

*Demographics*

Age, gender, and highest qualification attained (with the options: ‘Higher Education or professional / vocational equivalents’, ‘A levels or vocational level 3 or equivalents’, ‘GCSE / O Level grade A*‐C or vocational level 2 or equivalents’, ‘Qualifications at level 1 and below’, ‘Other qualifications: level unknown’, or ‘No qualifications’).

*Household members*

Participants will be asked about the members of their household, including the number of adults (aged 18+) in their household who have been included for consideration in the drink selection and the number of children (aged <18) in the household.

*Drinking behaviour risk*

The Alcohol Use Disorders Identification Test (AUDIT) (Bohn, Babor, & Kranzler, 1995) questions will be used to assess the level of risk associated with participants’ drinking behaviour.

*Weekly unit consumption*

Participants will be asked to enter the number of drinks they have consumed and purchased over the previous seven days, which will be used to calculate the number of alcohol units.

*Open text comment*

Participants will be asked to provide comments on task, such as explaining their choice of drinks, via an open text box to inform future studies in this area.

## Outliers

Any value deemed to be an outlier (defined by any values where the median absolute deviation exceeds 3) for the primary and secondary outcomes will be referred to the research team. If deemed to be a true value, any outliers will be excluded for a sensitivity analysis of that outcome.

## Missing data

Data that are not applicable will be coded as -888. Data that are missing due to an administrative or other error will be coded as -999.

#### Missing data checks

If an excessive amount of missing data (>10%) is identified for any variable, the research team will be notified so that checks can be made. If there is a sufficient amount of missing data for an outcome and there is not a valid reason for this (e.g. expected participant dropout for the purchasing outcomes), then it will be considered for exclusion. The missing status (yes/no) for these variables will be compared to demographic characteristics to assess if there was a systematic reason for missingness which might bias results.

## Violations of normality

If there is any indication of a strong departure from Normality for the residual QQ plots for any outcome model, a p-value and 95% confidence interval (CI) will be calculated using the bootstrap method from 1000 bootstrap samples – or a non-Gaussian regression approach considered.

## Descriptive statistics

A CONSORT flow chart will be constructed to show the numbers of participants assessed for eligibility, recruited, randomised, completed and analysed.

All raw outcome data will be reported in tables between the three study groups. Percentages will be reported for categorical variables, and the mean, median and standard deviation for continuous variables.

A table will also be produced which shows the number of participants that selected no drinks, no alcoholic drinks and no non-alcoholic drinks, between study groups (with a total column).

All additional measures will also be reported in a table to be sure they are adequately balanced between study groups.

## Statistical analysis

All analysis will be done in IBM SPSS version 27 or similar. Analysis will be coded in syntax and this will be added to a repository after analysis is complete.

All initial models listed below will include the two main effects of study group.

#### Covariates for all models

We will consider all additional measures for inclusion in each model as covariates (e.g. weekly unit consumption). The best fitted model, assessed through AIC (akaike information criterion), will be reported.

#### Primary outcome analysis

All randomised participants who complete the selection task will be included in the primary outcome analysis, including those who did not select any drinks and those that only selected non-alcoholic drinks (i.e., for these participants, their primary outcome, the number of alcohol units selected, will equal 0). This necessarily excludes any participants who fail to complete the selection task as well as those whose responses are flagged as incomplete or suspicious by the study team afterwards. For example, participants that select an unrealistically large number of drinks that are not purchased (e.g. over 100 drinks) or forge data (i.e. submit fake receipts for the purchasing outcome). The specific criteria for incomplete and suspicious participants will be developed during study monitoring and data cleaning.

A generalised linear model or similar - will be used to compare the primary outcome (the number of alcohol units selected) between the three study groups.

Each pairwise effect will be reported as a difference in means with 95% CI of the mean, t statistics and p-values. For the co-primary comparisons (using group 3 as the reference group), a 5%/2 adjustment to the significance threshold will be made. For the third comparison, where group 1 and 2 are compared, a 5%/3 adjustment to the significance threshold will be made. Cohen’s d (for each pairwise comparison) will also be calculated and presented alongside a 95% CI.

#### Secondary and additional outcome analysis

Secondary outcomes

1. Number of alcoholic drinks selected
2. Number of non-alcoholic drinks selected
3. Number of alcohol units purchased
   1. including additional drinks from study categories only
   2. including all additional drinks

Additional outcomes

1. Total number of drinks selected
2. Total number of drinks purchased
3. Number of alcoholic drinks purchased
   1. including all additional drinks
   2. including additional drinks from study categories only
4. Number of non-alcoholic drinks purchased
   1. including additional drinks from study categories only
   2. including all additional drinks

For the above outcomes, a generalised linear model, or similar, will be repeated as per the primary outcome model.

For the proportion outcomes (proportion of total drinks selected that are alcoholic and proportion of total drinks purchased that are alcoholic [a and b]) a binary logistic regression model, based on the counts of drinks, will be used to compare these outcomes between the three study groups. For these outcomes only, any participants who did not select a drink/ alcoholic drink/non-alcoholic drink (as appropriate for the outcome) will be excluded due to the nature of the model.

Each pairwise effect will be reported as a difference in means with 95% CI of the mean, t statistics and p-values. For the co-primary comparisons (using group 3 as the reference group), a 5%/2 significance threshold adjustment will be made. For the third comparison, where group 1 and 2 are compared, a 5%/3 significance threshold adjustment will be made. Cohen’s d (for all pairwise comparisons) will also be calculated and presented alongside a 95% CI.

For secondary and additional outcomes, results will be interpreted in light of the number of statistical tests conducted. We will not apply any formal correction for multiple comparisons (e.g., Bonferonni) as these are correlated outcomes, and such correction is therefore likely to be overly-conservative.

#### Tertiary analysis (Per-protocol selection and purchasing analyses)

Table 1. Participants included in two different analyses:

| **Participant included in:** | **Exact match of selected and purchased items** | **Additional drink purchases** |
| --- | --- | --- |
| Per-protocol analysis (1) | Yes | Either |
| Per-protocol analysis (2) | Yes | No |

#### Per-protocol analysis (1)– exact match of selected and purchased drinks, with additional drink purchases.

Only participants whose purchased products exactly matched their selected products, but who also did purchase other drinks, will be included for this per-protocol analyses of the primary outcome. This analysis will be the same as the primary outcome model above.

If there is a statistically significant effect for this outcome, then we will repeat this analysis for the equivalent purchasing outcome (‘number of alcohol units purchased’).

#### Per-protocol analysis (2)– exact match of selected and purchased drinks, with no additional drink purchases.

Only participants whose purchased products exactly matched their selected products, and who did not purchase any other drinks as instructed, will be included for this per-protocol analyses of the primary outcome. This analysis will be the same as the primary outcome model above.

#### In this analysis the purchasing outcome will map directly onto the selection outcome.
