# Supplementary material for: Impact on alcohol selection and online purchasing of changing the proportion of available non-alcoholic versus alcoholic drinks: A randomised controlled trial
Source: PLoS Med. 2023 Mar 30;20(3):e1004193. doi: 10.1371/journal.pmed.1004193 (PMC10062674; doi:10.1371/journal.pmed.1004193)
Supplement: Attachment — Submitted filename: Online Purchasing Avail. Reviewer Response FINAL.docx [file pmed.1004193.s005.docx]

Note:

Blue text is our response to reviewers

*italics is text that is dropped in from the paper, black italics is original text*, *red italics are our proposed changes*

Dear Dr. Clarke,

Thank you very much for submitting your manuscript "Impact on alcohol selection and purchasing of increasing the proportion of non-alcoholic versus alcoholic drinks: randomised controlled trial" (PMEDICINE-D-22-01553R1) for consideration at PLOS Medicine. 

Your paper was discussed with an academic editor with relevant expertise and sent to independent reviewers, including a statistical reviewer. The reviews are appended at the bottom of this email and any accompanying reviewer attachments can be seen via the link below:

In light of these reviews, we will not be able to accept the manuscript for publication in the journal in its current form, but we would like to invite you to submit a revised version that addresses the reviewers' and editors' comments fully. You will appreciate that we cannot make a decision about publication until we have seen the revised manuscript and your response, and we expect to seek re-review by one or more of the reviewers.  

In revising the manuscript for further consideration, your revisions should address the specific points made by each reviewer and the editors. Please also check the guidelines for revised papers at <http://journals.plos.org/plosmedicine/s/revising-your-manuscript> for any that apply to your paper. In your rebuttal letter you should indicate your response to the reviewers' and editors' comments, the changes you have made in the manuscript, and include either an excerpt of the revised text or the location (eg: page and line number) where each change can be found. Please submit a clean version of the paper as the main article file; a version with changes marked should be uploaded as a marked up manuscript.

In addition, we request that you upload any figures associated with your paper as individual TIF or EPS files with 300dpi resolution at resubmission; please read our figure guidelines for more information on our requirements: <http://journals.plos.org/plosmedicine/s/figures>. While revising your submission, please upload your figure files to the PACE digital diagnostic tool, <https://pacev2.apexcovantage.com/>. PACE helps ensure that figures meet PLOS requirements. To use PACE, you must first register as a user. Then, login and navigate to the UPLOAD tab, where you will find detailed instructions on how to use the tool. If you encounter any issues or have any questions when using PACE, please email us at [PLOSMedicine@plos.org](mailto:PLOSMedicine@plos.org).

We hope to receive your revised manuscript by Jul 04 2022 11:59PM. Please email us ([plosmedicine@plos.org](mailto:plosmedicine@plos.org)) if you have any questions or concerns.

***Please note while forming your response, if your article is accepted, you may have the opportunity to make the peer review history publicly available. The record will include editor decision letters (with reviews) and your responses to reviewer comments. If eligible, we will contact you to opt in or out.***

We ask every co-author listed on the manuscript to fill in a contributing author statement, making sure to declare all competing interests. If any of the co-authors have not filled in the statement, we will remind them to do so when the paper is revised. If all statements are not completed in a timely fashion this could hold up the re-review process. If new competing interests are declared later in the revision process, this may also hold up the submission. Should there be a problem getting one of your co-authors to fill in a statement we will be in contact.  YOU MUST NOT ADD OR REMOVE AUTHORS UNLESS YOU HAVE ALERTED THE EDITOR HANDLING THE MANUSCRIPT TO THE CHANGE AND THEY SPECIFICALLY HAVE AGREED TO IT. You can see our competing interests policy here: <http://journals.plos.org/plosmedicine/s/competing-interests>.

Please use the following link to submit the revised manuscript: 

<https://www.editorialmanager.com/pmedicine/>

Your article can be found in the "Submissions Needing Revision" folder. 

To enhance the reproducibility of your results, we recommend that you deposit your laboratory protocols in [protocols.io](http://protocols.io), where a protocol can be assigned its own identifier (DOI) such that it can be cited independently in the future. Additionally, PLOS ONE offers an option to publish peer-reviewed clinical study protocols. Read more information on sharing protocols at <https://plos.org/protocols?utm_medium=editorial-email&utm_source=authorletters&utm_campaign=protocols>

Please ensure that the paper adheres to the PLOS Data Availability Policy (see <http://journals.plos.org/plosmedicine/s/data-availability>), which requires that all data underlying the study's findings be provided in a repository or as Supporting Information. For data residing with a third party, authors are required to provide instructions with contact information for obtaining the data. PLOS journals do not allow statements supported by "data not shown" or "unpublished results." For such statements, authors must provide supporting data or cite public sources that include it. 

Please let me know if you have any questions, and we look forward to receiving your revised manuscript. 

Sincerely,

Richard Turner, PhD
Senior editor, PLOS Medicine

[rturner@plos.org](mailto:rturner@plos.org)

-----------------------------------------------------------

Requests from the editors:

Our academic editor suggested adopting somewhat more cautious language given the research design

We suggest the following amendment so that the title better matches journal style: "Alcohol selection and online purchasing with altered proportions of available non-alcoholic versus alcoholic drinks: A randomised controlled trial".

Thank you for this suggestion, which we like. We do also consider it important to make it clear in the first part of the title that we are actively manipulating the available options rather than simply observing the effect of different (altered) proportions. Therefore, we have now proposed a revised title that we think is still in line with the Editor’s suggestion, including removing the previous wording that had focused specifically on increasing the available non-alcoholic options. We are of course happy to discuss additional changes if our proposed title does not adequately address the Editor’s concerns.

*Impact on alcohol selection and online purchasing of changing the proportion of available non-alcoholic versus alcoholic drinks: A randomised controlled trial*

Please combine the "Methods" and "Results" subsections of the abstract.

These subsections have now been combined.

Please add a new final sentence to the new combined subsection, beginning "Study limitations include ..." and quoting 2-3 of the study's main limitations. 

We have now added study limitations to the Abstract (page 2, line 37-39).

*“Study limitations include the setting not being entirely naturalistic due to using a simulated online supermarket as well as an actual online supermarket, and that there was substantial dropout between selection and purchasing.*

Please quote the study dates in the abstract, and mention the country in which the study was done. 

Dates and country have now been added (page 2, line 10-11).

*“Adults (n=737) residing in England and Wales who regularly purchased alcohol online were recruited between March-July 2021.”*

Please also quote aggregate demographic details for study participants.

We have now added demographic details (mean age and gender split) to the Abstract (page 2, line 16).

*“607 participants (60% female, mean age = 38 years) completed the study and were included in the primary analysis.”*

In the abstract and throughout the text, please quote p values alongside 95% CI, where available.

We have now added p values in the Abstract and throughout the text.

After the abstract, please add a new and accessible "Author summary" section in non-identical prose. You may find it helpful to consult one or two recent research papers in PLOS Medicine to get a sense of the preferred style.

We have now added an “Author summary” section as follows.

***“Author summary***

*Why was this study done?*

*Decreasing the proportion of less healthy foods that are available - relative to healthier foods - reduces the likelihood of their selection.*

*A previous online study found that increasing the availability of non-alcoholic compared to alcoholic drinks increased their hypothetical selection, but there is an absence of evidence from naturalistic settings.*

*What did the researchers do and find?*

*This study evaluated the impact of increasing the proportion of non-alcoholic (relative to alcoholic) drinks, on selection and actual purchasing of alcohol. Participants were randomly assigned to one of three groups with varying proportions of alcoholic versus non-alcoholic drinks, and selected drinks in a simulated online supermarket before purchasing them in an actual online supermarket.*

*It was found that increasing the proportion of non-alcoholic drinks – from 25% to 50% or 75% - reduced the amount of alcohol selected and bought.*

*What do these findings mean?*

*This study provides evidence that increasing the proportion of non-alcoholic drinks could reduce alcohol selection and purchasing.*

*Further studies are warranted to assess whether these effects are realised in a range of real-world settings.”*

Please amend the heading of fig 1 to "Participant flowchart" or similar.

This has now been updated.

Please convert "18+", for example, to "aged 18 years and over", or similar.

This has now been updated (page 5, line 40).

Please substitute "sex" for "gender" where appropriate.

We have now substituted “sex” for “gender” (page 8, line 23; Table 1).

Please restructure the early part of the Discussion section (main text) so that the first paragraph is limited to summarizing the study's findings.

We have now restructured the Discussion so that the first paragraph discusses only the study findings (page 12, line 27-36).

Please avoid "the first" and similar claims, and where unavoidable add "to our knowledge" or similar.

We have now updated this throughout.

Throughout the text, please amend reference call-outs to the following style: "... interventions to food [7,8,10], ..." (noting the absence of spaces within the square brackets). 

In the reference list, please convert all italics to plain text. 

Where appropriate, please list 6 author names rather than 3, followed by 'et al.'.

Please remove all iterations of "[Internet]" from the reference list.

Please remove the information on data access, competing interests and funding from the end of the main text. In the event of publication, this information will appear in the article metadata via entries in the submission form, and does not need to be repeated here. 

Please include a completed CONSORT checklist with your revision, labelled "S1_CONSORT_Checklist" or similar and referred to as such in the Methods section (main text). 

In the checklist, please refer to individual items by section (e.g., "Methods") and paragraph numbers, not by line or page numbers as these generally change in the event of publication. 

We have now updated all of the above formatting points and included the CONSORT checklist.

Comments from the reviewers:

*** Reviewer #1: 

Thank you for the opportunity to review PMEDICINE 22-01553R1, Impact on alcohol selection and purchasing of increasing the proportion of non-alcoholic versus alcoholic drinks: randomized controlled trial. This study used a naturalistic randomized controlled trial to examine the impact of availability of alcoholic drinks and non-alcoholic drinks on consumers' alcohol selections. The authors report that increasing the proportion of drinks to be 75% non-alcoholic/25% alcoholic reduces alcohol selections compared to a 50%/50% and 25%/75% non-alcoholic/alcoholic product mix. The topic of the paper - strategies to reduce alcohol consumption - is highly important and timely given the growing burden of alcohol-related morbidity and mortality globally. The intervention tested is interesting, though its policy-relevance is not clear. Major strengths of the paper include the randomized design and the naturalistic online shopping task paired with actual shopping in an online
supermarket. I had concerns with the statistical analysis and potential policy relevance of the study, along with several more minor comments.

Major Concerns 
1.      The authors note that the primary outcome (and a number of the secondary outcomes) was highly skewed and zero inflated, and therefore chose to fit a hurdle model. Use of a hurdle model is appropriate; however, I was surprised to see that the authors state that they report in the Results "model results for positive values." This means that the primary results reported in the text exclude a substantial number of participants (those who didn't select any alcohol) and do not incorporate any potential effect of the interventions on probability of purchasing any alcoholic drinks. To my knowledge, this is not the standard way to report two-part models such as hurdle models. More typically, results from the two parts of the model are combined and a single marginal effect is reported (this can be accomplished using built-in commands in a variety of statistical packages or using bootstrapping). At the least, it is also not appropriate to focus the abstract and main text results on
just one part of the model and largely leave the results from the first part to the supplement, particularly given that the authors state that a very large number of participants buy no alcohol at all (i.e., outcomes are zero-inflated). From the results reported, we don't actually know to extent to which the intervention reduces overall drink purchasing, as the authors focus only on reductions in alcoholic drinks purchased among those who purchased any alcohol. That is an interesting outcome, but I do not think it is the outcome of most relevance to public health. I suggest the authors present combined marginal effects from the two parts of the model, rather than only focusing on effects among those who purchased any alcohol.

We thank the Reviewer for this suggestion and agree that it is clearer to present both parts of the model throughout (we originally did not include part 1 of the model in the Abstract to be as concise as possible). We have now added both parts of the model to the Abstract and for all outcomes to Table 3. We have also now calculated combined marginal effects from the two parts of the model and now report these in a new Table (Table 4), in the Abstract and throughout the Results.

Resulting changes to the Abstract are as follows (the same changes have been made to the Results section):

*“In the first part of a hurdle model, a greater proportion of participants in the ‘75% non-alcoholic’ group did not select any alcohol (13.1%) compared to the ‘25% non-alcoholic’ group (3.4%, p < 0.001); there was no evidence of a difference between the ‘50% non-alcoholic’ (7.2%) and the ‘75% non-alcoholic’ (p = 0.022) or ‘25% non-alcoholic’ (p = 0.121) groups. In the second part of a hurdle model in participants (559/607) selecting any drinks containing alcohol, the ‘75% non-alcoholic’ group selected fewer alcohol units compared to the ‘25% non-alcoholic’ (p < 0.001) and ‘50% non-alcoholic’ (p < 0.001) groups, with no evidence of a difference between the ‘50% non-alcoholic’ and ‘25% non-alcoholic’ groups (p = 0.178). Overall, across all participants, 17.46 units (95%CI 15.24, 19.68) were selected in the ‘75% non-alcoholic’ group; 29.4 units (95%CI 26.39, 32.42) in the ‘25% non-alcoholic’ group; and 25.51 units (95%CI 22.60, 28.43) in the ‘50% non-alcoholic’ group. This corresponds to 11.9 fewer alcohol units in the ‘75% non-alcoholic’ group compared to the ‘25% non-alcoholic’ group (a 41% reduction), and 8.1 fewer units (32% reduction) compared to the ‘50% non-alcoholic’ group; 3.9 fewer units (13% reduction) were selected in the ‘50% non-alcoholic’ group than in the ‘25% non-alcoholic’ group.”*

2.      I found the intervention interesting and appreciate the naturalistic randomized design used. I also appreciated that the authors are clear that the study largely offers a "proof of concept" that this type of intervention can affect consumer behavior. However, I was left wondering how important this proof of concept is given that consistent impacts were observed only for the condition that was very high in non-alcoholic drinks (75%). Although I agree that perhaps such change is in theory possible in a limited number of settings that explicitly cater to health-oriented consumers, it strikes me as wholly unrealistic for nearly all retail settings even in the medium term.  The authors do not indicate any policy levers are available for mandating such a change. I think the manuscript would be much stronger if the authors could provide a stronger rationale for why it might be feasible to expect such an intervention could be scaled to a meaningful degree.   

We agree with the Reviewer that a shift to 75% non-alcoholic drinks may not be realistic to apply across all retail settings, which is why we emphasise that the study is best defined as a proof-of-principle study. However, as we outline in the paper there are examples of settings that are not health-oriented that have made substantial shifts to make a higher proportion of healthier options available (e.g., the popular chain restaurant Wagamama’s have a 50% plant-based menu). We have also now added an additional example, which is a shift to 50% healthier options in healthcare settings in Scotland (GOV.UK, 2016). The increase in popularity of alcohol-free drinks is also relatively recent, with the global market growing substantially in the last 4 years (IWSR, 2022). In the UK, the low and no-alcohol market is forecast to continue to increase (Statistica, 2022) Therefore, although we would agree that this might not be realistic as an immediate policy recommendation, because popularity of these drinks continues to increase such changes are likely to become more realistic over time. It may also be that policies involve recommending a certain proportion of alcoholic vs. non-alcoholic options on display, rather than actively restricting alcoholic options, which may be less acceptable. These kinds of ‘availability’ interventions have been suggested to have potential at a policy level, but require more robust experimental evidence (Marteau et al, 2022). We think the current study provides such an initial step.

Irrespective of the above, we were relatively limited by resource in the current study, and this informed our decision to investigate a large shift in proportions of alcoholic vs non-alcoholic drinks, based on existing food studies that investigate similar variations in proportion and provide an indication of the likely effect (Pechey et al., 2022). Had we powered for smaller but potentially more achievable changes from a policy perspective, we may not have had adequate resource to conduct the trial. As we outline in the manuscript (page 14, line 13-15), future studies should build on this to investigate more granular changes in proportions. Similarly, in some analyses we do see some effects on behaviour with a smaller change in proportion, for example in the ‘50% non-alcoholic’ group (there was a lower percentage of total drinks that were alcoholic compared to the ‘25% non-alcoholic’ group.

We have now added further detail where we discuss the policy relevance of this paper in the Discussion (page 13, line 50 onwards).

*As it is yet to be seen if such major changes in ranges of drinks are feasible in real-world settings, these findings are most reasonably interpreted as proof of principle, rather than able to directly inform policy options. It is plausible that this situation could rapidly change, however. The increase in popularity of alcohol-free drinks is relatively recent, with the global market growing substantially in the last four years, and in the UK it is forecast to continue to increase [15]. This recent increase in the popularity of alcohol-free drinks has led to the emergence of drinking settings reflecting this, such as an alcohol-free off licence in London [35]. In food retail contexts there have been substantial changes seen in healthier or more sustainable ranges - such as the introduction of 50% plant-based menus [36] and the requirement to provide at least 50% healthier options in healthcare settings in Scotland [37] - suggesting that shifts of such magnitude are possible. However, before policy recommendations are made, a robust evidence base suggesting potential effectiveness is required [38], which this study provides an initial step towards.*

Minor comments:
3.      General: I found it very hard to remember what the condition names referred to. I suggest you rephrase the names of the conditions so that readers can immediately remember what they refer to vs. needing to refer back to the methods to remember that "High proportion" means High proportion of non-alcoholic drinks, i.e., 75% non-alcoholic/25% alcoholic drink mix.

We have now updated these names to a slightly shorter variation of this, where it is clearer what the condition names refer to.

*“75% non-alcoholic", 50% non-alcoholic", and "25% non-alcoholic”*

4.      Abstract: It is not clear what "model results" refers to here, as no modeling has been introduced. As noted above, I also do not think it is appropriate to only report results from half of the hurdle model.

We have now specified that hurdle models were used for analysis and have added both parts of the hurdle model to the Abstract.

*In the first part of a hurdle model, a greater proportion of participants in the ‘75% non-alcoholic’ group did not select any alcohol (13.1%) compared to the ‘25% non-alcoholic’ group (3.4%, p < 0.001); there was no evidence of a difference between the ‘50% non-alcoholic’ (7.2%) and the ‘75% non-alcoholic’ (p = 0.022) or ‘25% non-alcoholic’ (p = 0.121) groups. In the second part of the hurdle model in participants (559/607) selecting any drinks containing alcohol, the ‘75% non-alcoholic’ group selected fewer alcohol units compared to the ‘25% non-alcoholic’ (p < 0.001) and ‘50% non-alcoholic’ (p < 0.001) groups, with no evidence of a difference between the ‘50% non-alcoholic’ and ‘25% non-alcoholic’ groups (p = 0.178).*

5.      Introduction: It would be very helpful to have a sense of current availability of non-alcohol drinks in a typical supermarket or beverage store setting. It was hard to understand what the proportions tested in the trial mean without a sense of what proportion of beverages in the current marketplace are alcoholic vs. non-alcoholic. It would likewise be helpful to have a sense of current absolute sales of these drinks (you note that sales of non-alcohol drinks are increasing, but do not state the base level).

We thank the Reviewer for this suggestion and agree it is helpful information in order to interpret the findings. As part of our initial scoping work for the study we calculated the proportions of alcoholic beer and wine versus non-alcoholic alternatives currently available online at Tesco.com (alcohol-free beer, cider, or wine, or adult soft drinks). The proportions were approximately 25% non-alcoholic (360 options) and 75% alcoholic (1058 options). We have now added these proportions to the paper where we discuss our selection of drink options, in the Intervention section (page 6, line 32-34).

*Methods: Initial scoping work found that Tesco.com proportions of alcoholic versus non-alcoholic options were approximately 25% non-alcoholic (360 options) and 75% alcoholic (1058 options), based on the definitions used in the current study.*

In terms of the base level of non-alcoholic drink sales, they currently have a 3.5% volume share in the global alcohol industry (noting this is for no-and low-alcohol beer/cider, wine, spirits and ready-to-drink products) (IWSR, 2022). In the UK, low and no-alcohol beer accounts for 3% of the total beer market; this has doubled in the past 5 years (Financial Times, 2022) and is forecast to grow annually by 12.68% (Statistica, 2022) and is the fastest growing drinks segment in the UK. We have now added this detail on this to the Introduction (page 4, line 38-41).

For non-alcoholic soft drinks which made up half of our non-alcoholic drink options in the selection task, the market holds more than a 50% share in the global beverage market, although this includes all soft drinks such as coffee, tea, bottled water, fruit juices). Carbonated soft drinks make up just under 40% of the market (Fortune Business Insights, 2022).

*While the current market for alcohol-free beer, wine and spirits represents a 3.5% share of the global alcohol industry and is therefore relatively small, it is rapidly growing [13]. For example, low and no-alcohol beer currently accounts for 3% of the total beer market [14], but this is forecast to increase by nearly 13% per annum over the next 3 years and is the fastest growing drinks segment in the UK [15]. In 2021, the no/low alcohol market grew by 6% globally, and in the UK, sales of non-alcoholic beer increased by 7% [16].*

6.      Methods: It would be helpful to have a better understanding of the differences between the simulated online store and Tesco.com. For example, how many beverages does Tesco.com offer? What proportion of those drinks are alcoholic?

As highlighted in response to comment 5, Tesco.com offered similar proportions in their online store to the 25% non-alcoholic and 75% alcoholic group in our study which we have now highlighted in the manuscript (page 6, line 31-33), although we offered fewer options overall (64 in the current study compared to over a thousand options at Tesco.com). However, it would not have been feasible to match the number of options that Tesco.com had, and we offered a greater range of products than other studies of comparable ‘availability’ interventions (Blackwell et al., 2020; Pechey et al., 2022). We also chose a wide selection of different types of drinks, and were guided by brand popularity (see page 6, line 38), to ensure we had a wide range of options that were likely to be purchased. Participants often selected a wide variety of drinks, suggesting the drink options were sufficient for participants to be able to select a range of drinks that they wished to purchase.

We have now added further detail to the Methods to describe the drink options we selected and those available at Tesco.com (page 6, line 33-41).

*Initial scoping work found that Tesco.com proportions of alcoholic versus non-alcoholic options were roughly 25% non-alcoholic (360 options) and 75% alcoholic (1058 options). Alcohol-free beer, alcohol-free cider and alcohol-free wine options used in the task were matched as far as possible on brand and size characteristics with the alcohol options available online at Tesco.com. Additional alcoholic beer, cider and wine options were selected based on the leading brands of lager, ale, mild and stout, cider and wine [21–23] in Great Britain according to the number of consumers. Adult soft drinks were selected based on options that were commonly displayed next to alcoholic drinks in physical supermarkets and most likely to be consumed as a substitute for alcohol (meaning that children’s soft drinks, milk, and fruit juice were excluded).*

7.      Methods: It struck me that soft drinks might not be a typical substitute for alcoholic beverages. Studies of soda taxes, for example, find that soda and alcohol are not strong substitutes. Could the authors state why soft drinks were used as a non-alcoholic option? If soft drinks were appropriate here, why not have included a whole range of non-alcoholic beverages that consumers might regularly purchase, like milk, or 100% juice? Offering soft drinks feels like it could have enhanced the impact of the intervention over what might be expected in the real world, as it would provide consumers with number of drink options they might not typically see near the alcoholic drinks in a store.

We thank the Reviewer for this important point, however, there are some studies that do show that alcoholic drinks and soft drinks can be substitutes, for example one study found sustained increases in beer consumption following the implementation of SSB taxes in the US (Powell & Leider, 2022) and another found that changes in the price of soft-drinks changed purchasing patterns for alcohol, including beer, cider and wine, in the UK (Quirmbach et al., 2018).

We selected soft drinks that we defined as ‘adult soft drinks’ based on some initial scoping work in physical supermarkets where we found these drinks were often displayed near alcoholic beverages (e.g., soda water, tonic water, particular brands of soda) and not near drinks more likely to be consumed by children (e.g. fruit juices and brands of soda typically consumed by children such as Coca Cola, Fanta etc). We did not include milk, fruit juice or children’s soft drinks as we perceived these to be unlikely to be consumed by adults as a substitute for alcohol.

We also included a variety of alcohol-free beer, cider, and wine in each of the non-alcoholic selections, with equal proportions of these types of drinks versus soft drinks in every group. Although these drinks may be perceived as more of a direct substitute for alcoholic beverages, there were not enough alcohol-free options available at Tesco.com to include only these options in the majority non-alcoholic group, and we also found that soft drinks were selected more frequently by participants.

We have now added some further details on how we selected these non-alcoholic drink options (page 6, line 33-40).

*Alcohol-free beer, alcohol-free cider and alcohol-free wine options used in the task were selected based on brand and size matches, where possible, with alcohol options available online at Tesco.com. Additional alcoholic beer, cider and wine was selected based on the leading brands of lager, ale, mild and stout, cider and wine in Great Britain according to the number of users. Adult soft drinks were selected based on options that were commonly displayed next to alcoholic drinks in physical supermarkets and most likely to be consumed as a substitute for alcohol (i.e., children’s soft drinks, milk, and fruit juice were excluded).*

8.      Methods: Suggest including in the main text a few more details on how drinks were chosen for the simulated online store. The supplement makes it clear that considerable thought went into choosing these products - a few of these details in the main text would be helpful. Likewise, I suggest indicating that the products were shown in random arrangement in the main text (vs. only mentioning in the supplement).

We have now added some further detail on the drink choices to the Methods, from the supplement and in response to Point 7 (page 6, line 33-40). We have also added further details on the randomisation (page 6, line 47-49).

*Participants were randomised to the order in which each subcategory (soft drinks; alcohol free; alcoholic) was presented within each of the beer and wine categories; the order of drinks within each subcategory was also randomised.”*

9.      Methods: Suggest you provide more details on what instructions where given to participants for each part of the experiment. (E.g., the text states that participants were shown instructions, but does not provide details on exactly what participants were asked to do).

We have now added further detail to the Methods on what instructions were given to participants (page 7, line 15-20).

*“Participants were initially provided with an information sheet, instructions, and a link to the study via email. Participants were told the study was investigating “Adult drink preferences in England and Wales” and were not made aware of the study aim. Participant instructions outlined the stages of the study in detail, i.e., that participants were required to select the drinks for their next shop from Tesco.com in a simulated online supermarket (Stage 1), then to immediately go to Tesco.com to book a delivery slot and add these drinks to their shopping basket (Stage 2), and finally to send their receipt to the study team on their delivery or collection day (Stage 3). Once they had started the study task, participants were again presented with this information and provided consent.”*

10.     Methods:  You might consider adding a sentence that explicitly differentiates "units" from "drinks." I realize you define "units" in the primary outcome paragraph, but the secondary outcomes all sound so similar to one another that it was hard to remember the difference between "drinks" and "units," especially because "drinks" was never defined. Perhaps my confusion was partly because "drinks" can often mean "standard drinks" (at least in the US context) and units can often refer to individual products (e.g., "unit sales") regardless of how many standard drinks the product contains. Another idea would be to use the word "standard drink" instead of "unit," which (at least in the US context) might be more familiar to readers.

We thank the Reviewer for pointing this out. We have now made clear when we define units that we refer to UK units and have noted the equivalent in US standard drinks as well as adding a reference (Cleavebooks, 2022) (page 7, line 47). We have also added the standard drink equivalent to the Results section where we first mention units and as a footnote to each of the tables.

*In the UK, a unit is a standard measure of pure alcohol in a drink with one unit equivalent to 10ml or 8g of pure alcohol (this is equivalent to 0.56 of a US standard drink [25]).*

11.     Statistical analyses: Why were different thresholds set for the different comparisons? How were these levels selected vs. a more standard adjustment? (E.g., Bonferroni-Holm, Benjamini-Hochberg)

A two-stage adjustment procedure was adopted because the two comparisons to the ‘25% non-alcoholic’ group were of primary interest (treated as co-primary contrasts, *p*=5%/2) and the additional comparison between the remaining two groups (‘75% non-alcoholic’ and ‘50% non-alcoholic’) was of tertiary interest, therefore a more standard adjustment procedure was not possible. A conservative adjustment of 5%/3 was used rather than a more standard method which would require no longer having control over which p-values are reported. For example the Benjamini-Hochberg or Bonferroni-Holm procedures require choosing an arbitrary false discovery rate, and this means we would no longer have control over which p-values are reported.

We have clarified this in the paper (page 9, lines 29-35).

*For all outcomes, for the co-primary comparisons of primary interest (using the ‘25% non-alcoholic’ group as the reference group), a 5%/2 adjustment to the interpretation threshold for statistical significance was made. For the third comparison of tertiary interest (where ‘75% non-alcoholic’ and ‘50% non-alcoholic’ groups were compared), a simplistic 5%/3 adjustment was made rather than using methods which may not report all p-values (e.g. Benjamini-Hochberg, Holm- Bonferroni). These additional tests were calculated by refitting the same model just with different reference categories*

12.     Statistical analyses: How were participants who did not buy any drinks (either alcoholic or non-alcoholic) treated in each of the analyses?

The outcomes are real numbers including zero, which is why modelling of zero inflation was used. We have clarified this in the paper by adding further detail to the Methods section (page 9, line 3).

*The data included participants that did not select any drinks, as this was still a valid choice.*

13.     Results, throughout: I found the results reporting confusing - it appears as though the model was used to calculate differences between groups in counts, but then the 95% CI's in the text are reported in in terms of proportional changes. It's not clear where these CIs come from - suggest clarifying in the analysis section or reporting CIs in the same units as the coefficients were estimated (i.e., counts). This applies throughout (including the abstract).

We now report the marginal effect estimates and their associated 95%CIs, along with the reduction as the overall reduction in predicted counts (marginal effect estimates) and the associated % reduction. We no longer report the proportional changes for part 2 of the model or the 95% reduction around these changes.

14.     Page 8 - Primary outcomes, "there were no differences in zero values for the other comparisons" - suggest rephrasing for clarity, e.g., to "there were no differences in likelihood of selecting no alcoholic drinks containing alcohol."

We have now rephrased this sentence (page 10, line 6-10).

*In the first part of the hurdle model, a greater proportion of participants in the ‘75% non-alcoholic’ group did not select any alcohol (13.1%) compared to the ‘25% non-alcoholic’ group (3.4%, p < 0.001); there was no evidence of a difference in the ‘50% non-alcoholic’ (7.2%) and the ‘75% non-alcoholic’ group (p = 0.022, given the adjusted threshold of p = 0.0167) or the ‘25% non-alcoholic’ group (p = 0.121).*

15.     Page 8-9, Purchasing: It was surprising that the Higher proportion group selected a lower proportion of alcohol drinks than the lower proportion group (43% vs. 60%) and that the Lower proportion and Same proportion groups differed (60% vs. 56%) but the difference between the Higher proportion group  and the Same proportion group was not significant (43% vs. 56%), despite being much larger in magnitude than the difference between Lower and Same groups. The 95% CI's reported do not overlap (which I understand is not the same as the difference between the groups being significant, though often when CIs do not overlap, there is a significant difference). Could the authors speculate on why this pattern of results might have occurred?

We previously reported the average percentage for this outcome, which does not relate directly to the distribution of the data. However, we have now calculated marginal effects which we report for these outcomes instead in Table 4. These more clearly map onto the significant differences between groups.

16.     Page 9, Per Protocol Analysis - I did not quite understand what this meant: "Of the 422 participants who purchased drinks, 344 participants purchased the drinks they had selected in the selection task (with 78 participants missing one or more drinks); 182 participants purchased exactly the drinks they had selected with no additional drinks." I think this means that the 344 purchased the drinks they selected and also additional drinks and that 78 of those 344 had missing drinks, but it was not totally clear.

We thank the Reviewer for highlighting this and agree it is confusing, we have now updated this sentence to make this clearer (page 11, line 37-40).

*Of the 422 participants who purchased drinks, 344 participants purchased all the drinks they had selected in the selection task and 78 participants had one or more missing drinks. Of the 344 participants that purchased all the drinks they selected, 182 purchased no additional drinks.*

17.     Page 9, Per Protocol Analysis - The authors note that although attrition was greater among participants with higher baseline alcohol purchasing, "modeling suggested this did not bias comparison between groups." This finding is crucial to the study interpretation, so I would suggest describing the results of the modeling in more detail, so readers can assess for themselves the evidence for/against biased attrition, without having to consult the supplemental materials. Additionally, in the supplemental table S4B, it's clear that the interaction between drinks and remained/lost is significant for nearly all results, especially when using Model A. This should be addressed in both the results and the discussion of the main text.

We have now added further detail of the modelling in the main paper (page 11, line 42-47). Additionally, in the supplementary material we report two models to assess whether there was an interaction between attrition and baseline alcohol purchasing: Model A and Model B. Model A results had poor diagnostics, whereas Model B did not, which means the results from Model B give the best indication of whether there is an interaction. We realise that it is confusing to report both; therefore, we now only report the model with acceptable diagnostics.

*Chi-squared tests indicated that there was no evidence against assuming equal attrition occurred. Exploratory analyses indicated that attrition was greater amongst participants with higher baseline alcohol purchasing, but regression using an interaction term suggested this did not bias the comparisons between groups, as there was no evidence of an effect at the usual threshold for interaction terms (p=0.01) (Supplementary Material S4).*

18.     Table 3. It was unclear what the coefficients reported for part 1 of the hurdle model referred to. I expected to see differences in predicted probability of selecting any alcohol, or perhaps an odds ratio, but it appears as if counts are reported. I would also suggest adding a table note describing the modeling in a bit more detail and clarifying where each estimate comes from (e.g., for the primary outcome, it was not clear where the 95% CIs for the proportional reductions come from for part 2 of the model given that a count model was used, and likewise it took me a minute to understand what the p-values refer to).

We now report the marginal effect estimates and their associated 95%CIs, along with the reduction as the overall reduction in predicted counts (marginal effect estimates) and the associated % reduction. We no longer report the proportional changes for part 2 of the model or the 95% reduction around these changes.

19.     Figure 1: For Group 1, 1 participant was excluded from analysis for not meeting drinking inclusion criteria…Shouldn't this person have been excluded prior to enrolment/randomization?

Participants were asked a screening question by the research agency when they were initially invited to take part in the study, and the response to this screening question indicated they were eligible (i.e., that they consumed alcohol weekly). Participants were then asked a number of questions about their drinking during the study and it was in these questions that the participant gave inconsistent responses and indicated that they did not consume alcohol.

We have now re-worded this in Figure 1 for clarity.

20.     Figure 1: In the allocation boxes, I would suggest keeping the n's for "did not receive full intervention" in same bullet point as their explanation (vs. 2 separate bullet points)

We thank the Reviewer for pointing out this typo. We have now updated this, so they are in one bullet point.

21.     Figure 2: Do you have any data on the participants who dropped out in the selection task? I realize the dropout rate is not very high, and does not appear to be highly differential by arm (though was nearly 2x as high in the 50/50 alcoholic-non-alcoholic arm), but if you have any screening data on these participants, it might be useful to see whether the people who dropped out in each arm were roughly similar to one another

Thank you for this suggestion, which we agree would be useful to know. We did contact the research agency to see if it was possible to get details on these participants. However, as the study is complete the participant files are now archived. The agency has stated that, although it is possible to unarchive the data, as we did not originally outline that we would need these details from them initially, to access them would be resource intensive and would incur costs. Unfortunately, we do not have the resource to do this. In future studies we will collect these details on participants from the agency initially so we can examine any potential differences in dropout.

22.     Supplemental S2: Some n's are missing in the column headings.

We have now added the missing n’s.

23.     Supplemental S4B: I did not understand what was being reported in this table - suggest adding additional table notes, particularly given that these analyses are not described in detail in the main text. Additionally, it's not clear what "poor diagnostics" means in this context. This feels especially important given that the interaction was significant for most Model A results. 

We had previously reported both models in the supplementary material as we considered Model A may be easier to interpret but had poor model diagnostics. However, upon reflection we realise this is potentially confusing to interpret. Therefore we now only report the more trustworthy model that has acceptable diagnostics (Model B) and no longer report Model A (see point 17).

*** Reviewer #2: 

Thank you for the opportunity to review this paper, which examines the impact of the proportion of alcohol drinks on alcohol units selected. The research question is valuable and the topic is timely given the high public health burden of alcohol consumption. However, my enthusiasm for the research is limited mostly by concerns about the external validity of the simulated "store."   

Major concerns:
*       The authors describe the simulated "store" as a naturalistic setting but it appears to be images of products mocked up in Qualtrics. In my opinion, this protocol does not feel very realistic and no data are provided about the external validity of the platform. I have questions such as: have the authors used this protocol before? Does behavior in the Qualtrics store correlate with actual shopping behavior? What did the "store" look like to participants? (I didn't see any images in the paper). Were prices displayed? Were there any elements of a real online store like price promotions, banners, etc.? What kinds of information about the products were available within the simulated store? (e.g., could participants view nutrition information about the products?)

We thank the Reviewer for this comment and agree that it would be useful to have more details on how the store looked in the paper. The purpose of the simulated store was not to directly mimic Tesco.com in its entirety, as we were unable to recreate certain aspects (e.g., the inclusion of the copyrighted Tesco logo; price promotions). Instead, the aim of the simulated supermarket was to display a selection of drinks in an online setting where products and information given about the products mapped as closely as possible to those same products at Tesco.com. This enabled the purchasing task to be used to validate the selection outcome, linking selection to a meaningful consequence (albeit imperfectly), and we could therefore include a more objective measure of shopping behaviour than selection alone. We did still attempt to make the simulated supermarket look as naturalistic as possible within Qualtrics: we used similar font and colour themes and included the drinks in a similar layout. This enabled participants to add drinks to their basket by clicking on each drink and receive a total basket cost once they had made their selection. In terms of product information, we included the name of the drink (matching the Tesco.com definition exactly), the % ABV for drinks containing alcohol and the price of each drink. We did not include nutritional information as we did not want to include too much information on the selection page and it was not possible to click on a product to find additional information. Participants were, however, provided with links to all the products at Tesco.com so they were able to find out this information should they wished to.

We have updated the Methods to include more detail on the characteristics of the simulated store and product details (page 5, line 31-34 and page 6 line 50). We have been informed that including images of the task in the paper would necessitate obtaining copyright approvals, but we have linked to them via the OSF project page.

*Methods: The study was conducted online using simulated and real online supermarkets. First, participants completed a simulated supermarket selection task hosted on the Qualtrics online survey platform (see* [*https://osf.io/2cy7t*](https://osf.io/2cy7t) *for example task images). The simulated supermarket was designed to look and function as similarly to the actual online supermarket as was possible within Qualtrics. Drinks were displayed in rows of four drink options and participants could add these to their basket, which displayed a total price after the selection had been made.*

*Each drink option was displayed as an image, below which was a text description of the drink (identical to Tesco.com), the %ABV for drinks containing alcohol, and its price.*

We also found that purchasing and selection mapped closely onto each other. It is possible that this is because our instructions required participants to purchase the drinks they selected and we do not know how this might map onto real behaviour. However, this is arguably true for many experimental studies where participants are behaving, such as in selecting or purchasing products, whilst being aware they are participating in a study. More generally, simulated online supermarkets are commonly used to measure the impact of such interventions on purchasing behaviour (Hartmann-Boyce et al., 2018; Payne Riches et al., 2019), and our study is likely more externally valid than purely hypothetical settings given the inclusion of an actual purchasing measure. However, we do agree that further studies should evaluate the external validity of the platform that is used and have added this to the Discussion (page 13, line 32).

*However, to our knowledge, this is the first study of an availability intervention to make use of such a setting (albeit in conjunction with a simulated supermarket component), although simulated supermarkets are commonly used in similar intervention studies [32.33]. This represents the most robust design used to date and could provide a useful method through which to assess interventions without requiring complex collaboration with commercial retailers, although further research is needed to assess its external validity.*

*       I appreciate that the authors attempted to heighten the realism of the protocol by having participants "purchase" their selected drinks in a real store. However, I am not convinced that this element of the protocol really addresses the concerns with external validity because the real store did not reflect their experimental arm (proportion of alcoholic drinks) and many more products were available than in the initial task. Ultimately, the protocol felt a bit clunky and hard to follow and the case for doing the second task felt fairly weak to me.

The principal aim of the purchasing task was to make the selection in the simulated store as realistic and meaningful as possible (i.e., to encourage participants to select drinks that they actually intended to purchase), rather than to have a real store directly reflect the experimental arm. This is one of the reasons that selection was our primary outcome, as it was assessed in the same context that the intervention was applied (e.g. see page 8, line 2). We agree that ideally we would have only had one stage, and assessed purchasing directly in the setting where the intervention was implemented, but this was not feasible with our available resource and without an established partnership with a major retailer. We agree that the current design does have its limitations, which we hope have been addressed in the Discussion, but as far as we are aware it is the most robust design to date to assess this type of intervention. Additionally, as this is the first study to use this method, future research using the same procedure could refine the process, for example by taking further steps to minimise attrition and providing clearer instructions to ensure participants do not purchase additional drinks, so that the purchasing outcome more directly maps onto and validates the selection outcome. We have now added detail to the paper to make it clearer that the main purpose of the purchasing stage was to strengthen the selection outcome, rather than to provide a separate context in which the intervention is assessed for a second time (page 13, line 21-24).

*Drinks were initially selected within a simulated online supermarket, before purchasing was completed in an actual online supermarket (albeit with the visual presentation of the former modelled on the latter). The principal purpose of including a measure of purchasing in the actual online supermarket was to validate and strengthen our primary outcome of selection, rather than to measure purchasing behaviour in a separate context. However, this meant that additional drink options were available in the real online supermarket, and participants could not be prevented from buying these if they wished to.*

*       For the power analysis - I wondered why the authors did not attempt to estimate a range of plausible effect sizes based on research on product availability from other domains (e.g., food). As written, the study feels exploratory in nature.

We decided not to estimate a power calculation based on previous effect sizes, as there was insufficient directly relevant literature to inform this (particularly from alcohol studies) and we were limited by resource. However, we did use existing literature to generate and pre-specify clear hypotheses and analytic intentions, and so we would disagree that the study is mainly exploratory in nature.

We have added more detail to the sample size section to give more context on effect sizes found in previous studies and to explain why these were not directly used for the calculation (page 6, line 1-10).

*A previous online study compared the impact on drink selection of altering non-alcoholic vs alcoholic drink availability (ref). The proportion of participants selecting an alcoholic drink decreased from 74% when non-alcoholic drink availability was low (25% of drink options), to 61% when availability was medium (50% of drink options), and 51% when availability was high (75% of drink options) (i.e., a difference of 13% and 10%, respectively, between adjacent groups). However, only a single drink was selected in this online study and there was no intention to purchase the selected drinks (i.e., decisions were purely hypothetical). As such, to our knowledge there was no comparable evidence available from which to estimate effects of this intervention on selection or purchasing behaviour of multiple drink options ~~with the intention to purchase~~. ~~As this was the first trial of this intervention to assess alcohol selection and purchasing of multiple drink options, no comparable evidence was available from which to estimate effects.~~ Available resources allowed recruitment of around 600 participants. As an illustrative calculation, assuming 15% attrition, a sample of 510 participants (170/group) was sufficient to detect an effect of d=0.3 for the primary outcome for a two-group t-test with alpha of 5% and 80% power. Using pre-testing data (~5/group), the conservative SD estimate was 12.1 (i.e., the maximum group variance observed), indicating that the sample size was sufficient to detect a difference of 3.7 alcohol units selected between groups.*

*       The authors briefly comment on the fact that some of the non-alcoholic options contained sugar. I would encourage the authors to more thoroughly address the potential unintended consequences of people switching from alcohol to sugar-sweetened beverages given different but substantial health risks to drinking SSBs.

We agree with the Reviewer that interventions to reduce SSB consumption are equally important as those to reduce alcohol consumption, to ensure that alternative options to alcohol are as healthy as possible (i.e., zero or lower sugar). The ‘adult soft drinks’ that were included in our non-alcoholic options were generally much lower in calories than standard soft drinks (such as Coca-Cola and Fanta, which we excluded due to being marketed to both adults and children rather than as an alternative to alcohol). Although some non-alcoholic options contained sugar, the mean number of calories across our non-alcoholic drink options was much lower than the alcoholic options (e.g., 64 vs 233, respectively, in the ‘50% non-alcoholic’ group) and included options such as soda water with no calories or sugar. Drinks that contain alcohol are also often exempt from SSB interventions, so there is likely to be more existing regulation of sugar and calories in non-alcoholic drinks (IAS, 2020), as well as transparency for consumers given the requirement to provide calorie information on labels, from which alcohol is exempt.

Additionally, no and low alcohol alternative to alcoholic drinks contain fewer calories and less sugar than full strength drinks (Nicholls, 2022). However, there is currently no policy on how the marketing of these drinks should be guided from a public health perspective - the alcohol industry commonly market these products as healthier than they are and self-regulation is inconsistent (Corfe et al., 2020). We therefore highlight in the paper that this could carry potential harms and needs to be monitored closely (page 14, line 33) and we have added detail to highlight the potential harms of SSB consumption (page 14, line 49-42).

*It is important to note that because many alcohol-free alternatives are marketed by the alcohol industry and there is no regulation on the often-exaggerated health claims that are made about these drinks [41]. Such industry involvement has potential harms and should be monitored closely [42–44]. In addition, although some of the non-alcoholic drink options in the current study contained no sugar and were generally lower in calories than the alcoholic options (an average of 64 calories per non-alcoholic drink versus 233 calories per alcoholic drink), many soft drinks and alcohol-free alternatives still contain large amounts of sugar and calories [45]. Given the health risks associated with sugary drink consumption [45], continued regulation and policies to reduce sugar content and consumption from both alcoholic and non-alcoholic drinks is needed to mitigate these risks.*

Other comments
*       I wondered how the authors arrived at the ranges of the proportions they manipulated (75%, 50%, 25%)?

As highlighted in the response to Reviewer 1 (point 2). We used proportions in line with proportions commonly used in the existing availability literature. For example the same proportions were used in a previous online alcohol selection study (Blackwell et al., 2020). Food studies have also used similar proportions (Pechey et al., 2022). We have now added these references (page 6, line 45).

*The proportions used were based on previous food and alcohol studies [12,24].*

*       More information on how the authors picked the drink options would be helpful. Was this based on any data reflecting popular choices / market share / sales?

We had included details on the drink option selection in the supplementary material, but have now added further detail to the Methods (see responses to point 7 and 8, Reviewer 1) in response to this comment and comments from Reviewer 1.

*       More detail on the scalability of this intervention would be useful in the intro and discussion. Is this intervention something that retailers might do voluntarily? If so, is there precedent for stores doing this? Or is this intervention something that would be required as a policy at the local or federal level? (and similarly if so, is this on the table as part of policy discussions/plans anywhere?)

As we highlight in response to comment 2 from Reviewer 1, we describe the study as proof of concept as we acknowledge that such changes to the current availability of alcoholic drinks might not be realistic to suggest as a potential policy change until there is more evidence for their effectiveness in a variety of settings (see page 14, line 14). In line with this, a recent analysis piece on availability interventions suggests we need more systematic evidence in a form accessible to policymakers (Marteau et al., 2022). Despite this, there are some examples of availability implementations, such as in Scotland, where all retail outlets in healthcare buildings must provide 50% of food and 70% of drink options that are not high in fat, sugar or salt (GOV.UK, 2016). There are also current proposals to significantly increase the availability of alcohol free and low alcohol products in the UK by 2025 (DHSC, 2019). Both these examples indicate there is scope for potential policy implementation. There are also examples in the food industry of businesses changing the availability of healthier or more sustainable food options voluntarily, such as Wagamama’s shift to 50% plant-based options (Vegconomist, 2021 page 14, line 9). However, we acknowledge that it may be unlikely that these changes will be driven by businesses, as they could potentially fear a loss of revenue, although it has been highlighted that such losses are not generally reported in evaluations (Marteau et al., 2022). It has been recommended that businesses that rely heavily on selling unhealthy items should be supported by governments to provide healthier alternatives (Marteau et al, 2022).

We have now added further examples and details on the potential scalability of this intervention to the Discussion (page 14, line 5-13).

*This recent increase in the popularity of alcohol-free drinks has led to the emergence of drinking settings reflecting this, such as an alcohol-free off licence in London [35]. In food retail contexts there have been substantial changes seen in healthier or more sustainable ranges - such as the introduction of 50% plant-based menus [36] and the requirement to provide at least 50% healthier options in healthcare settings in Scotland [37] - suggesting that shifts of such magnitude are possible. However, before policy recommendations are made, a robust evidence base suggesting potential effectiveness is required [38], which this study provides an initial step towards.*

*       Were any of the analytic decisions not pre-registered? Outlining deviations / modifications to the protocol (if any) would be helpful. For example, the switch to the hurdle model seems like it was not pre-specified based on my review of the OSF plan.

There were no deviations from the protocol or the statistical analysis plan. There was one exploratory analysis that was not pre-registered to investigate any differences in attrition across groups (Supplementary Analysis S4), which we have now labelled as exploratory (page 11, line 39). The decision on excluded data was also not pre-registered as we had not collected similar data before and were therefore not aware of the issues with participants following the protocol that we would encounter. We have now highlighted this and added further detail on when the criteria were applied to the Statistical Analysis section (page 8, line 50).

In the statistical analysis plan we stated that generalised linear models or similar would be used for analysis and a hurdle model is a type of generalised model. We did not state the specific model used ahead of data collection as the characteristics of the data were used to guide the choice of model (e.g., we did not predict that the data would be zero inflated).

*       I would suggest including raw percentages in the text of the results in the primary outcome section for the part I of the hurdle model.

We have now added these to the Abstract and main Results.

*       Does the p-value adjustment use a specific approach? What is the rationale for dividing 5%/2?

Please see our response to comment 11 from Reviewer 1: a two-stage adjustment procedure was adopted because the two comparisons to the ‘25% non-alcoholic’ group were of primary interest (treated as co-primary contrasts, p=5%/2) and the additional comparison between the remaining two groups (‘75% non-alcoholic’ and ‘50% non-alcoholic’) was of tertiary interest, therefore a more standard adjustment procedure was not possible. A conservative adjustment of 5%/3 was used rather than a more standard method which would require no longer having control over which p-values are reported. We have clarified this in the paper (page 9, line 29-35).


*** Reviewer #3: 

The article is more cautious now, and I recommend publication

We thank the Reviewer for their endorsement of the manuscript.

*** Reviewer #4: 

Overall, I found the manuscript clear and easy to follow. I have only have minor comments listed below.

I note that no adjustment was made for multiplicity (3 arms) in the sample size calculation; however, according to the SAP (hypothesis testing section), the two main comparisons are set using alpha of 2.5%

The sample size statement now makes clear that the sample size was primarily based on pragmatic considerations (i.e., time and money) given there was no direct evidence for an effect size and that the calculation is an illustrative one. Therefore, we have clarified the statement to now say “As an illustrative calculation…”.

Please confirm that the criteria used to exclude data (e.g. incomplete/suspicious data or forged receipt) was defined and applied while unaware of the group allocation.

We can confirm that these criteria were defined during data collection and while researchers were unaware of group allocation. We have now added this detail to the statistical analysis section (page 8, line 50).

*“Participants who failed to complete the selection task and those whose responses were flagged as incomplete or suspicious - e.g., those that forged data (i.e., submitted fake receipts) or selected an unrealistically large number (e.g., over 100) of drinks that were not purchased – were excluded (see Figure 1 for details by group). The criteria used to exclude data were not pre-registered but were defined and applied prior to data analysis, while researchers were unaware of group allocation.”*

The SAP pre-specifies that a generalised linear model would be used. I understand the issue of skewness and excess of zero which led to the use of a two-part model; however, I would be keen to see the results using a linear model including all data (i.e. including zeros) as initially planned. This could be reported in the supplement. 

We have not reported results with any other modelling approach as these are inadequate for handling the observed excess of zeros. The current hurdle model is a type of generalised linear model.

I find Table 3 difficult to read. I would recommend adopting the following format: mean difference (95% CI), p=0.xxx. For example: -0.64 (-1.44; 0.17)  p=0.121. The same applies to Table 4 and tables in the supplement.

Thank you for this suggestion - we have now updated to this format in all tables.

When reporting outcome results in the text, please report the main results as mean differences instead of percentage changes. In Tables, please also consider removing the reporting of results as percentage change. Percentage changes could potentially be reported in the supplement only and used in the discussion to assist with the interpretation.

We have now reported these in the Abstract and Results as reductions in marginal effect estimates. However, we have still included the percentage change alongside these reductions to aid interpretation and because we judged it was confusing to introduce these for the first time in the Discussion. We have removed percentage changes from all the tables.

When reporting the results for the primary outcome please consider first reporting the proportion of participants selecting at least one alcoholic drink (part 1 of the model) before reporting the results of part 2.

We have now updated our Results to report part 1 of the model first.

422/640 (66%) of participants went on to purchase drinks. Are the reasons for not proceeding to the purchasing stage known? If so, please consider reporting them. It would also be interesting to further understand whether the profiles of participants differ according to whether or not they went on to purchase drinks. Pease compare the characteristics (both baseline measures and purchasing intention) of participants who carried on with the purchase vs those who did not. In case of meaningful differences, please consider additional sensitivity analyses.

Unfortunately, we were unable to collect the reasons for participants not continuing, most participants stopped responding to reminders to complete the next stages and it was therefore not possible to obtain any details on why they did not continue. We do report that those who did not continue with the study had higher baseline alcohol purchasing compared to those that continued (page 11, line 43).

*“Exploratory analyses indicated that attrition was greater amongst participants with higher baseline alcohol purchasing, but regression using an interaction term suggested this did not bias the comparisons between groups, as there was no evidence of an effect at the usual threshold for interaction terms (p = 0.01)”*

I understand that the analysis models were unadjusted. I would suggest performing sensitivity analyses adjusted for key prognostic factors such as alcohol consumed the previous week (or alcohol purchased and the previous week or AUDIT score).

Alternative models with additional covariates were considered, and they tended to be i) worse fitting based on their AIC values and ii) led to non-convergent models due to lack of sample size. Some models where covariates were warranted (i.e., that included baseline consumption) are reported.

References

Alcohol Health Alliance. (2022). NoLo product placement in soap operas: Alcohol health promotion or alibi branding? *Alcohol Health Alliance*. https://ahauk.org/nolo-product-placement-in-soap-operas-alcohol-health-promotion-or-alibi-branding/

Blackwell, A. K. M., De-loyde, K., Hollands, G. J., Morris, R. W., Brocklebank, L. A., Maynard, O. M., Fletcher, P. C., Marteau, T. M., & Munafò, M. R. (2020). The impact on selection of non-alcoholic vs alcoholic drink availability: An online experiment. *BMC Public Health*, *20*(1), 526. https://doi.org/10.1186/s12889-020-08633-5

Cleavebooks. (2022). *Equivalent Unit Drinks Calculator*. http://www.cleavebooks.co.uk/scol/ccalcoh1.htm

Club Soda. (2021). Club Soda opens London’s first alcohol-free off-licence. *Club Soda*. https://joinclubsoda.com/first-alcohol-free-off-licence/

Corfe, S., Hyde, R., & Shepherd, J. (2020). Alcohol-free and low-strength drinks. *Social Market Foundation*, 95.

DHSC. (2019). *Advancing our health: Prevention in the 2020s – consultation document*. GOV.UK. https://www.gov.uk/government/consultations/advancing-our-health-prevention-in-the-2020s/advancing-our-health-prevention-in-the-2020s-consultation-document

Financial Times. (2022). UK sales of low-alcohol and no-alcohol beers almost double in 5 years. *Financial Times*.

Fortune Business Insights. (2022). *Non-alcoholic Beverages Market Size, Share | Industry Analysis [2027]*. https://www.fortunebusinessinsights.com/industry-reports/non-alcoholic-beverages-market-101927

Gov.uk. (2016). *Criteria for the Healthcare Retail Standard*. http://www.gov.scot/publications/criteria-healthcare-retail-standard-1/

Hartmann-Boyce, J., Bianchi, F., Piernas, C., Payne Riches, S., Frie, K., Nourse, R., & Jebb, S. A. (2018). Grocery store interventions to change food purchasing behaviors: A systematic review of randomized controlled trials. *The American Journal of Clinical Nutrition*, *107*(6), 1004–1016. https://doi.org/10.1093/ajcn/nqy045

IAS. (2020). Reformulate sugary alcohol drinks. *IAS*. https://www.ias.org.uk/news/reformulate-sugary-alcohol-drinks/

IWSR. (2022). *No- and Low-Alcohol in Key Global Markets Reaches Almost US$10 Billion in Value*. IWSR. https://www.theiwsr.com/no-and-low-alcohol-in-key-global-markets-reaches-almost-us10-billion-in-value/

Marteau TM, Hollands GJ, Pechey R, Reynolds JP, Jebb SA. (2022). Interventions changing the assortment of available food and drink for leaner, greener diets: Under-specified and under-used? *BMJ*.

Mialon, M., & McCambridge, J. (2018). Alcohol industry corporate social responsibility initiatives and harmful drinking: A systematic review. *European Journal of Public Health*, *28*(4), 664–673. https://doi.org/10.1093/eurpub/cky065

Nicholls, D. E. (2022). *The Marketing and Consumption of No and Low Alcohol Drinks in the UK*. 51.

Payne Riches, S., Aveyard, P., Piernas, C., Rayner, M., & Jebb, S. A. (2019). Optimising swaps to reduce the salt content of food purchases in a virtual online supermarket: A randomised controlled trial. *Appetite*, *133*, 378–386. https://doi.org/10.1016/j.appet.2018.11.028

Pechey, R., Hollands, G. J., Reynolds, J. P., Jebb, S. A., & Marteau, T. M. (2022). Is altering the availability of healthier vs. less-healthy options effective across socioeconomic groups? A mega-analysis. *International Journal of Behavioral Nutrition and Physical Activity*, *19*(1), 88. https://doi.org/10.1186/s12966-022-01315-y

Petticrew, M., Maani, N., Pettigrew, L., Rutter, H., & Van Schalkwyk, M. C. (2020). Dark Nudges and Sludge in Big Alcohol: Behavioral Economics, Cognitive Biases, and Alcohol Industry Corporate Social Responsibility. *The Milbank Quarterly*, *98*(4), 1290–1328. https://doi.org/10.1111/1468-0009.12475

Powell, L. M., & Leider, J. (2022). Impact of the Seattle Sweetened Beverage Tax on substitution to alcoholic beverages. *PLoS ONE*, *17*(1), e0262578. https://doi.org/10.1371/journal.pone.0262578

Quirmbach, D., Cornelsen, L., Jebb, S. A., Marteau, T., & Smith, R. (2018). Effect of increasing the price of sugar-sweetened beverages on alcoholic beverage purchases: An economic analysis of sales data. *Journal of Epidemiology and Community Health*, *72*(4), 324–330. https://doi.org/10.1136/jech-2017-209791

Standard drink. (2022). In *Wikipedia*. https://en.wikipedia.org/w/index.php?title=Standard_drink&oldid=1097896461

Statistica. (2022). *Non-Alcoholic Beer—United Kingdom | Statista Market Forecast*. Statista. https://www.statista.com/outlook/cmo/alcoholic-drinks/beer/non-alcoholic-beer/united-kingdom

Vegconomist. (2021). *Wagamama Confirms 50% of Menu Will Be Plant-Based By October - vegconomist: The vegan business magazine*. https://vegconomist.com/gastronomy-and-catering/wagamama-confirms-50-of-menu-will-be-plant-based-by-october/
