# Supplementary material for: Impact on alcohol selection and online purchasing of changing the proportion of available non-alcoholic versus alcoholic drinks: A randomised controlled trial
Source: PLoS Med. 2023 Mar 30;20(3):e1004193. doi: 10.1371/journal.pmed.1004193 (PMC10062674; doi:10.1371/journal.pmed.1004193)
Supplement: Attachment — Submitted filename: Online Purchasing Avail _Reviewer response _FINAL 20230201.docx [file pmed.1004193.s006.docx]

**Impact on alcohol selection and online purchasing of changing the proportion of available non-alcoholic versus alcoholic drinks: A randomised controlled trial**

**Response to Editorial comments**

ABSTRACT

1. Line 16: “…16 purchasing. 607 participants (60% female, mean age = 38 years)…” please include the age range

We have now added the age range for participants (page 2, line 16).

2. Line 17: “. In the first part of a hurdle model, a greater proportion…” suggest reporting the results followed by the statistical method used to derive them, “ A greater proportion….as demonstrated by the first part of…” similarly for line 21

We tried changing the order as suggested, by reporting the results first. However, there was author consensus that the original wording was clearer and more readable so we would prefer to keep this passage as currently formulated if possible.

3. Thank you for including p-values and confidence intervals. Please define CI at first use.

We have now defined CI at first use (page 2, line 19).

4. Throughout the abstract (and main text) p-values are presented for some outcome measures and confidence intervals for others (in the main text CIs are absent – see below). Please ensure that for all outcome measures both p-values and 95% CIs are reported suggest placing CIs within square parentheses as follows: “(95% CI [15.24, 19.68], p= or p<0.001)

We have now updated the Abstract and Results section and included p-values alongside 95% CIs where possible. The only results we were unable to do this for were the marginal effect estimates as there are no p-values generated for such estimates. A marginal effect estimate is the average effect (value predicted from the model) for one variable, at the average value of each of the other variables. These 95% CI are conditional on these average values and are from our existing models. Therefore no new modelling has been done. The R package used to produce these has been referenced in Table 4 (Fox, 2003).

We have reported confidence intervals as we typically do and have done in a recent paper published in PLOS Medicine e.g. (95% CI 15.24, 19.68; p < 0.001) (see Hollands et al., 2022, <https://doi.org/10.1371/journal.pmed.1003920>) as we found this simpler and clearer to read given it involves less punctuation. However, if formatting within square parentheses is now a journal requirement, then we are of course happy to update them e.g. (95% CI [15.24, 19.68]; p < 0.001).

5. When reporting p-values please report as <0.001 (not <.001) and where higher please report actual values as p= 0.12, for example, not p= .12 Please check and amend throughout including the main manuscript text, tables and figures, where relevant

We have now updated p-values to the recommended format throughout the manuscript.

AUTHOR SUMMARY

6. Thank you for including an author summary.

It’s rather difficult to appreciate exactly what you did just by reading the author summary alone and this should not be the case.

Line 6 onwards – it might be helpful to include some of the details you report early in your introduction to give context to the importance/necessity of the study

Line 15 onwards - detail is missing which explains clearly to the reader what you did, how you did it and how doing it is helpful. Please revise accordingly - some specific points are detailed below.

Please include the study design and number of participants “In a randomised controlled trial of X number of ppts…” for example, or something similar

It may be beneficial to the reader to further elaborate here a) on the simulation part of the study, what was simulated, what did participants look at? suggest explaining briefly what “availability” interventions are to give context and clarity and b) separately describe what participants did in the actual supermarket and how the two events are connected, as written its not clear, a lot is left to assumption (or knowledge of detail contained within the main manuscript)

Line 16: at “Participants…” please make into a separate bulleted point

Line 21: Additional detail of your findings would be helpful here also – reducing the amount of alcohol how and when

Line 26: suggest including an additional point which states the implications of this i.e. why is that helpful?

We have now updated our author summary to include more detail based on these helpful suggestions (page 3, lines 8-40)

INTRODUCTION

7. Line 47: Suggest removing/substituting the word “Although…” or at line 46

We have now removed ‘although’ from the start of this sentence (page 4, line 48).

METHODS and RESULTS

8. When reporting p-values please report as <0.001 (not <.001) and where higher please report actual values. Please check and amend throughout including the main manuscript text, tables and figures, where relevant

We have now updated p-values throughout the manuscript.

9. For all outcome measures please report p-values and 95% CIs, as detailed above

Please see our response to point 4 above. We have now updated the Abstract and Results section and included p-values alongside 95% CIs where possible.

10. Please define %ABV at first use (line 49 was the 1st I could find but please check)

We have now defined %ABV at first use (page 6, line 49).

TABLES

11. Please ensure that each table is affiliated to an appropriate caption/legend which clearly details the table contents, without the need to refer to the main manuscript text. Please check and amend throughout including in the supplementary files

We have now ensured that each table (in the manuscript and supplementary file) has an appropriate and more detailed caption and legends to clearly describe table contents.

12. Please ensure that p-values as well as 95% CIs are reported in all tables. In the table captions/legends where p-values are reported please define the test used to derive them and the significance level

We have included p-values along with 95% CIs where possible. We have not added them to the marginal effect estimates table (Table 4) as they are not possible to calculate (see our response to point 4). We have now added the tests used to derive the p-values to the relevant legends along with relevant references: marginal effect estimates (Fox, 2003); hurdle models (Brooks et al., 2017); beta binomial models (Crowder, 1978; Prentice, 1986).

13. In the table titles please ensure that the abbreviation CI is defined

We now define CI in all table titles.

14. Please report p as <0.001 (not .001) or if higher the exact p-value as p = 0.14 (not .14), for example

We have now updated this throughout the tables.

DISCUSSION

15. Please remove all sub-headings from the discussion, including the conclusion such that it reads as a single piece of continuous prose

We have published two papers in *PLOS Medicine* in the past calendar year that have included such subheadings in their Discussion sections (Hollands et al., 2022, https://doi.org/10.1371/journal.pmed.1003920; Reynolds et al., 2021, https://doi.org/10.1371/journal.pmed.1003743) which did not receive any comment. If it is at all possible, we would therefore prefer to keep them in as we do think the use of these subheadings significantly helps the structuring of the discussion to aid the reader. Of course, if this is a new and binding formatting requirement that did not apply previously, we are happy to abide with it and remove them throughout the Discussion.

16. Page 12, line 26: Suggest tempering this statement “Our data show that

We have now updated this sentence as suggested (page 12, line 33).

17. Page 12, 37: “The findings of the current study are in line with…” suggest “consistent with…”

We have now updated this sentence (page 12, line 44).

18. Page 12, line 45: Please add “to the best of our knowledge...” or something similar

We have now updated this sentence (page 13, line 2).

19. Page 14, line 46: Suggest “To our knowledge, …” or “This randomised controlled trial is the first, that we are aware of, to assess…” or something similar

We have included ‘to our knowledge’ in this sentence (page 15, line 4).

REFERENCES

20. Please check the bibliography to ensure that journal name abbreviations are those found in the National Center for Biotechnology Information (NCBI) databases.

We have now updated all our references to ensure the journal abbreviations are those found in the NCBI database.

SOCIAL MEDIA

To help us extend the reach of your research, please provide any Twitter handle(s) that would be appropriate to tag, including your own, your coauthors’, your institution, funder, or lab. Please enter any handles you wish to be included into the relevant part of the manuscript submission form.

@BehavChangeDsgn, @GJHollands, @Clarke_NC1, @MarteauTM, @MarcusMunafo, @JennFerrar, @kdeloyde, @MarkAPilling

Comments from Reviewers:

Reviewer #1: Thank you for the opportunity to review the revised manuscript, Impact on alcohol selection and online purchasing of changing the proportion of available non-alcoholic versus alcoholic drinks: A randomised controlled trial. The authors have responded fully and thoughtfully to each of my comments. I have no further questions.

Reviewer #2: I thought the reviewers were very responsive to comments. No further comments from me!

Reviewer #4: My comments have been adequately addressed. It is good to see both parts of the models reported together with the marginal effects. I believe it makes the interpretation much clearer.

-Laurent Billot

Any attachments provided with reviews can be seen via the following link:

__________________________________________________

In compliance with data protection regulations, you may request that we remove your personal registration details at any time. (Use the following URL: <https://www.editorialmanager.com/pmedicine/login.asp?a=r>). Please contact the publication office if you have any questions.

**References**

Brooks, M. E., Kristensen, K., van Benthem, K. J., Magnusson, A., Berg, C. W., Nielsen, A., Skaug, H. J., Mächler, M. and Bolker, B. M. (2017). glmmTMB balances speed and flexibility among packages for zero-inflated generalized linear mixed modeling. The R Journal, 9(2), 378–400.

Crowder, M.J., 1978. Beta-binomial anova for proportions. Applied Statististics. 27, 34-37.

Fox J (2003). Effect Displays in R for Generalised Linear Models. *Journal of Statistical Software*, **8**(15), 1–27. https://doi:10.18637/jss.v008.i15.

Hollands, G. J., Usher-Smith, J. A., Hasan, R., Alexander, F., Clarke, N., & Griffin, S. J. (2022). Visualising health risks with medical imaging for changing recipients’ health behaviours and risk factors: Systematic review with meta-analysis. *PLOS Medicine*, *19*(3), e1003920. https://doi.org/10.1371/journal.pmed.1003920

Prentice, R.L., 1986. Binary regression using an extended beta-binomial distribution, with discussion of correlation induced by covariate measurement errors. J.A.S.A. 81, 321-327

Reynolds, J. P., Ventsel, M., Kosīte, D., Dames, B. R., Brocklebank, L., Masterton, S., Pechey, E., Pilling, M., Pechey, R., Hollands, G. J., & Marteau, T. M. (2021). Impact of decreasing the proportion of higher energy foods and reducing portion sizes on food purchased in worksite cafeterias: A stepped-wedge randomised controlled trial. *PLOS Medicine*, *18*(9), e1003743. https://doi.org/10.1371/journal.pmed.1003743
